# Age Effects in L2 Grammar Processing as Revealed by ERPs and How (Not) to Study Them

**DOI:** 10.1371/journal.pone.0143328

**Published:** 2015-12-18

**Authors:** Nienke Meulman, Martijn Wieling, Simone A. Sprenger, Laurie A. Stowe, Monika S. Schmid

**Affiliations:** 1 Center for Language and Cognition, University of Groningen, Groningen, The Netherlands; 2 Department of Humanities Computing, University of Groningen, Groningen, The Netherlands; 3 Centre for Research in Language Development throughout the Lifespan (LaDeLi), Department of Language and Linguistics, University of Essex, Colchester, United Kingdom; Leiden University, NETHERLANDS

## Abstract

In this study we investigate the effect of age of acquisition (AoA) on grammatical processing in second language learners as measured by event-related brain potentials (ERPs). We compare a traditional analysis involving the calculation of averages across a certain time window of the ERP waveform, analyzed with categorical groups (early vs. late), with a generalized additive modeling analysis, which allows us to take into account the full range of variability in both AoA and time. Sixty-six Slavic advanced learners of German listened to German sentences with correct and incorrect use of non-finite verbs and grammatical gender agreement. We show that the ERP signal depends on the AoA of the learner, as well as on the regularity of the structure under investigation. For gender agreement, a gradual change in processing strategies can be shown that varies by AoA, with younger learners showing a P600 and older learners showing a posterior negativity. For verb agreement, all learners show a P600 effect, irrespective of AoA. Based on their behavioral responses in an offline grammaticality judgment task, we argue that the late learners resort to computationally less efficient processing strategies when confronted with (lexically determined) syntactic constructions different from the L1. In addition, this study highlights the insights the explicit focus on the time course of the ERP signal in our analysis framework can offer compared to the traditional analysis.

## Introduction

When it comes to learning a second language (L2) in a naturalistic setting, comparisons of younger and older starting learners consistently show an advantage for those who arrived in the L2 country earlier in life (e.g. [1]). In particular, many aspects of grammatical structure can remain problematic for later learners despite extended periods of immersion [2]. Yet, it is unclear how the language-learning ability develops across the lifespan, as evidenced by the ongoing debate about the dynamics of the age effect and its continuous or discontinuous development [3]. The present study explores the age effect on learners’ brain responses as measured by event-related potentials (ERPs) and provides further support for the use of a novel statistical framework to do so.

Knowledge about the true shape of the age curve could inform theories of language learning [4]. Yet, our present knowledge of the dynamics of the age curve is incomplete, in particular where the acquisition of morphosyntactic features is concerned. Results of behavioral studies (such as grammaticality judgments, sentence-picture verification and elicited production) investigating the influence of age of acquisition (AoA) on L2 grammatical performance, are to some extent contradictory (see e.g., [4–10]), even in replication studies (see the replications of [6] by [9] on the one hand and [11] on the other). Partly, this is a methodological problem: since behavioral measures do not only reflect grammatical processing but can also be contaminated by metalinguistic knowledge, decision making and other strategic processing, interpreting the results can be difficult.

In the present study we therefore make use of ERPs to investigate online neural responses during sentence processing. ERPs, which are extracted from the EEG signal, are known to be fast, unconscious, and highly sensitive to linguistic anomalies [12], and can therefore inform us about the underlying neural and cognitive processing mechanisms that occur without conscious control. Specifically, we investigate age effects on two aspects of grammatical processing, an area for which strong claims have been made for age-related changes in L2 learning.

In first language (L1) processing, two primary types of ERP components have been associated with grammatical processing: the P600 and the left anterior negativity (LAN). The P600 has consistently been found in response to the processing of errors in morphological and other syntactic information in native speakers (e.g., [12–15]). This component has been elicited under extremely heterogeneous conditions, and although it is often believed to be associated with a late stage of (re)analysis of information [12,16], its exact functional interpretation remains debated [17–19]. There is, however, a very strong correlation between the appearance of the P600 effect and grammatical violations. With respect to the presence of the LAN, the findings are more varied [20]. This component has been associated with morpho-syntactic agreement processes [15,20,21], but it has also been claimed that it is a more general index of working memory load [18,22]. Because the appearance of a LAN is variable, even in native speakers, our primary focus in the current study will be on the P600.

In research on second language processing, the development of L2 proficiency, particularly in the initial stages of learning, has been associated with large changes in ERP signatures. For example, in response to grammatical violations a gradual shift has been reported with increasing proficiency from an N400 in novice learners to a P600 in more proficient ones [23,24]. In L1 speakers, the N400 is typically associated with semantic processes (see [25] for an overview). These results are therefore thought to reflect a shift in processing strategies, from a reliance on statistical dependencies to the induction of a generalized rule. That is, the P600 as a violation marker indicates whether a specific aspect of the L2 has been grammaticalized.

However, even after years of exposure, at the end stage of L2 acquisition (often referred to as ‘ultimate attainment’), learners’ ERP signatures can still deviate from those of native speakers. Weber-Fox and Neville [26] were among the first to investigate this effect. They investigated the processing of violations in different syntactic structures (i.e. phrase structure, specificity constraint) in five groups of Chinese learners of English, with an AoA ranging from 1 to >16. A LAN was reported in all early L2 groups, but was more broadly distributed for learners who were exposed between the ages of 11 and 13 and absent in the group that acquired the L2 after the age of sixteen. The subsequent P600 was comparable to monolinguals up to an AoA of 10 years, but was delayed in latency for an AoA between 11 and 13 years and absent in later learners (AoA >16 years). The authors conclude that “maturational changes significantly constrain the development of the neural systems that are relevant for language” (p. 231).

Apart from the Weber-Fox and Neville study, AoA effects have not been systematically studied with ERPs. Instead, an all-or-nothing approach has usually been taken in which a group of (late) L2 learners is compared to a group of native speakers. A study by Hahne [27] corroborated the findings from Weber-Fox and Neville, in the sense that in her group of late Russian learners of German (AoA > 10), the LAN seen in native speakers in response to phrase structure violations was absent and the subsequent P600 was delayed. Recent studies show a more diverse pattern of results. Whereas some (late) learner groups show very similar ERP patterns compared to natives (e.g., [28,29]), others show delayed, reduced or even entirely absent ERP components [29–33]). Taken together, the available literature does not allow firm conclusions about the effect of AoA on ERPs. This is due to the fact that both the target structures under investigation and the language groups being compared vary, resulting in varying degrees of L1-L2 similarity (a topic which will be discussed in more detail below). Another issue concerns the proficiency of the L2 speakers [34]. More centrally to the current study however, the AoA ranges for the various L2 groups differ between investigations, making it impossible to reliably infer the shape of the proficiency curve.

The effect of AoA on the ERP signal measured during L2 grammar processing thus remains unclear. Yet, ERPs could provide us with vital information about how neural mechanisms are impacted by delays in L2 acquisition. This could be of particular interest to debates on the ‘critical period hypothesis’. The concept of a critical period is understood as a language-related maturationally constrained window of opportunity, ending at some point around the end of childhood, after which learners are not able to reach native-like proficiency in the L2. Proponents have found support for this assumption in some areas of phonology and morphosyntax for adult/late L2 learners compared to young/early L2 learners or natives (e.g., [5–7]), while others claim that these age effects can be explained without a critical period for language acquisition since they are due to confounds with other factors that impact L2 learning (e.g., [4,8–10]).

**Fig 1:**
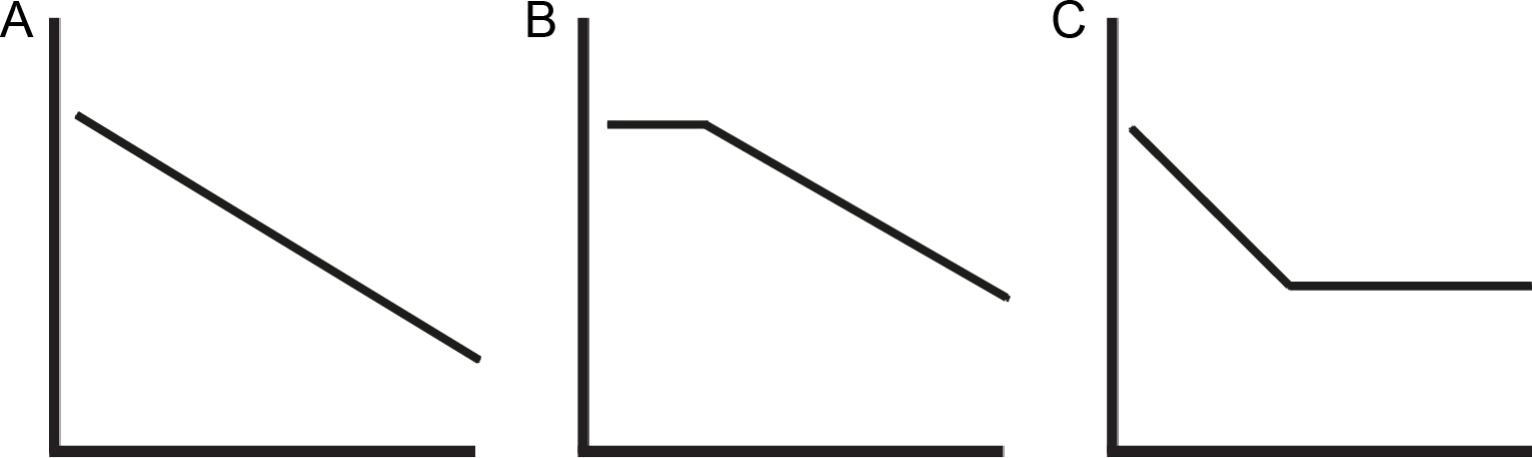
Various age curves. Schematic representations of L2 proficiency (vertical axis) declining over AoA (horizontal axis). Only Panels B and C support a potential ‘critical period’, because of the presence of a discontinuity in the AoA function. Figure taken from [36].

As Birdsong [4,35,36] points out, there are specific geometric and temporal features the age curve needs to meet in order to qualify as evidence for a critical period in L2 acquisition. More specifically, the function that relates AoA to attained L2 proficiency should not be linear, but instead should contain a discontinuity. As such, age effects in the form of Panels B and C of Fig 1 would qualify as evidence for potential maturational constraints on language acquisition, whereas Panel A would reflect the effects of more general factors, such as cognitive development and competition from L1, which exert their effects during the entire lifespan. However, as Birdsong notes, it is often difficult to assess such characteristics of the shape of the curve, because of a lack of granularity in studies of age effects.

This is clearly the case for the majority of ERP studies discussed above, which typically only include a group of late L2 learners (AoA >12) and do not treat AoA as a continuous variable. There are several problems associated with this approach, as has been amply demonstrated in behavioral studies (see [37]). First, binning of age groups is highly arbitrary and results in a loss of information as to age effects within these ‘bins’. As a result, a significant difference between two (arbitrary) age groups can be driven by a subset of individuals rather than the group as a whole. Second, and even more importantly, categorical group comparisons make the investigation of the dynamics of the age curve impossible.

**Fig 2:**
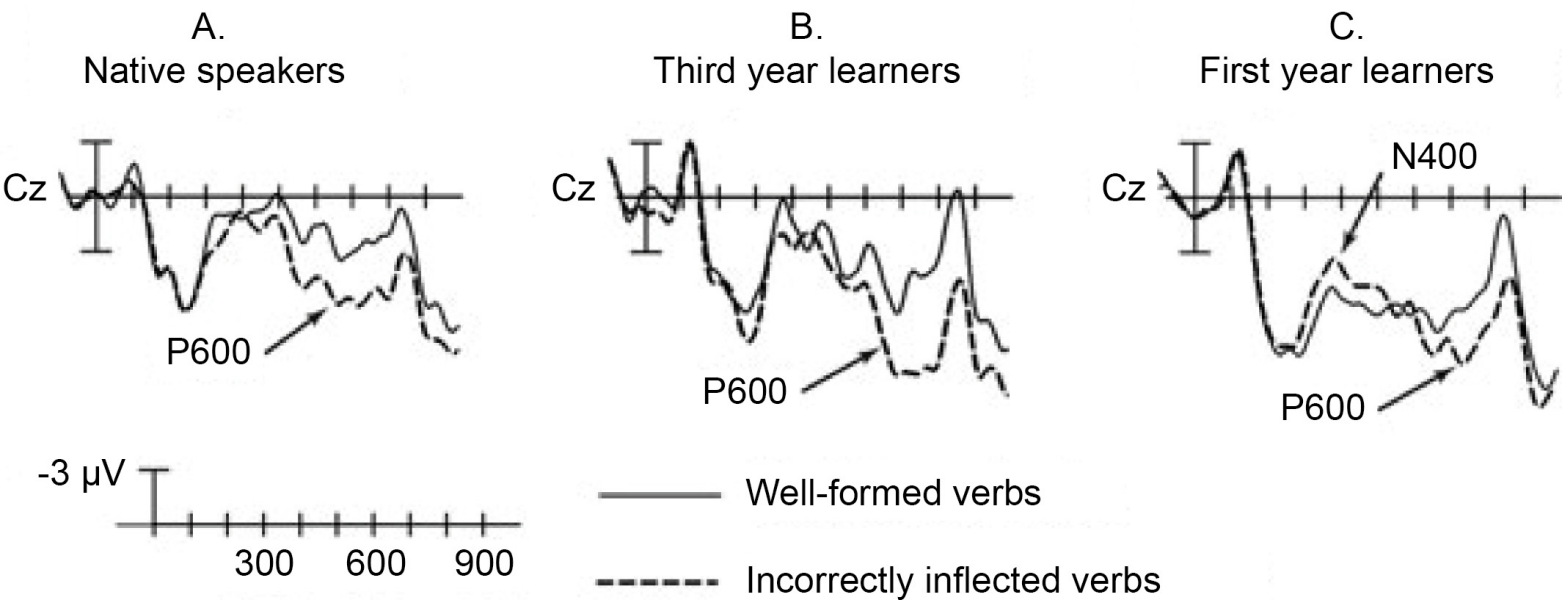
Group differences in multiple aspects of the ERP signal. An example of how ERP signatures can differ between groups (in this case native speakers, beginning and intermediate L2 learners). We see variation in form, latency and amplitude of parts of the ERP wave across these groups. It is difficult to objectively capture these differences in a traditional analysis, which is often artificially limited to time windows. Figure taken from [23].

If we want to study the effect of AoA on L2 acquisition, the methodological concerns described above hold for virtually any form of performance measurement. ERPs present additional problems, because they can vary in a number of different dimensions, including the form of the response, its latency and its amplitude (see Fig 2). All of these may vary with age in interesting ways. However, these complex interactions cannot be adequately investigated using the standard statistical tools.

In this study we therefore make use of generalized additive modeling (GAM) to investigate the ERP signal. As GAMs have not yet been used frequently to study ERP data (with the exception of [38–42]), we provide a brief introduction to this method. An in-depth discussion of the GAM method can be found in [43].

**Fig 3:**
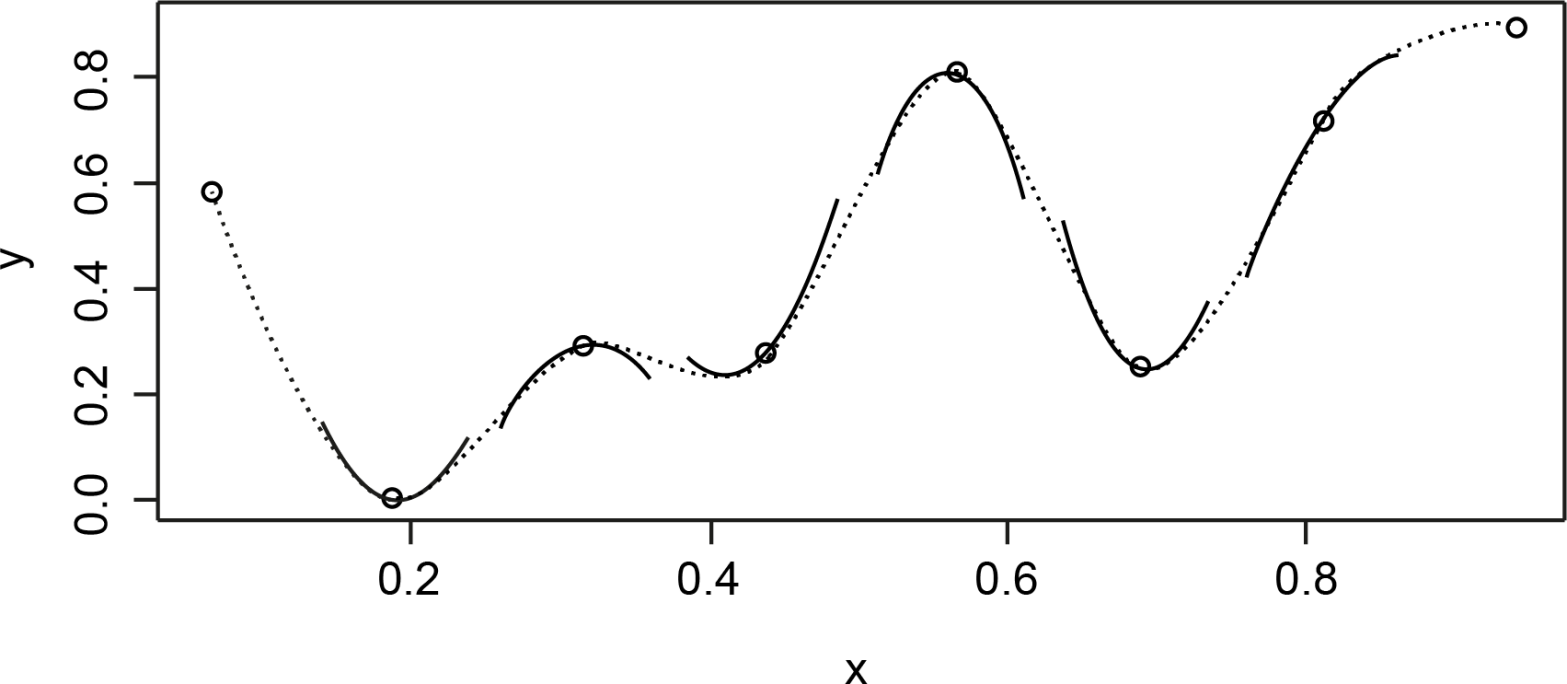
Example of a basis function: a cubic regression spline. A cubic regression spline is a non-linear curve constructed from sections of cubic polynomials joined together so that the curve is continuous. The cubic regression spline shown (dotted curve) is made up of seven sections of cubic polynomials (solid lines). The points at which they are joined (and the two end points) are known as the ‘knots’ of the spline. Figure taken from [43].

A generalized additive model is an extension of a generalized linear regression model in which non-linear terms (or non-linear interactions between terms) can be included besides the (standard) linear relationships between predictors and the dependent variable. GAMs are therefore much more flexible than simple linear regression models. They do not require a predefined non-linear function specification, but rather determine the non-linear function (i.e. smooth) automatically, in any number of dimensions. Specifically, the non-linearities are modeled by so-called basis functions. For example, a cubic regression spline models a non-linearity by connecting several third-degree polynomials (see Fig 3). The places where the polynomials are connected are called ‘knots’, and the higher the number of knots available for the non-linearity, the more complex the non-linearity is allowed to be. Another type of basis function, is the thin plate regressions spline which models the non-linearity by combining several low-level functions (such as a linear function, a quadratic function, a logarithmic function, etc.). The complexity of this type of non-linearity is limited by the number of low-level functions which are combined in the smooth. As the thin plate regression spline is the best approximation of the non-linear pattern [44], this is the basis function we use in this study.

In a GAM, multiple predictors may be combined in a single smooth, yielding essentially a wiggly surface (when two independent variables are combined) or a wiggly hypersurface (when three or more independent variables are combined). The estimation processes that determine the smooth functions and parameters are designed to avoid overgeneralization and overfitting by employing cross-validation.

Because of their ability to detect non-linear effects, GAMs are particularly well-suited to analyzing the ERP signal. An important focus in ERP analysis is the time course of the ERP effects (i.e. the period after the critical region of the stimulus has been presented and during which linguistic processing unfolds). Due to the large size of ERP datasets, it has become standard practice to average the signal in a predefined time window of interest. This may not only lead to a loss of power, but also means that possible latency differences between individuals can no longer be studied. The GAM approach resolves these issues by assessing the complete (non-linear) effect of time on the ERP signal. Fortunately, recent improvements [45] have allowed GAMs to cope with very large datasets, such as EEG data. As a regression method, numerical variables can be investigated as predictors without resorting to group dichotomization. Furthermore the GAM approach also allows for the inclusion of random effects, and thereby enables one to appropriately deal with item variation and to model imbalanced data, a common situation in ERP studies.

In the present study we therefore use GAMs to examine the effect of AoA on the ERP signal in L2 learners, such that we meet Birdsong’s [4] criteria for an appropriately fine-grained investigation of age effects. We present a detailed analysis of the full variability of the ERPs: We include AoA as a continuous variable, and look at the (non-linear) ERP pattern over time in order to examine potential latency effects, while simultaneously accounting for subject and item variation. To illustrate the potential benefits of using GAMs compared to a more traditional approach, we first present an ANOVA and a correlational analysis of the ERP data. Subsequently, a GAM analysis of the same dataset will be presented. We will show that the GAM approach provides unique insights into the dynamics of the influence of AoA on the ERP signal, which cannot be obtained by the traditional approach.

We investigate age effects on grammatical processing in Slavic (Polish and Russian) speakers with advanced L2 proficiency in German and a large range in AoA, and compare these results to those of a group of native German controls. We presented our participants with auditory sentences with correct and incorrect instances of two constructions: non-finite verbs and grammatical gender agreement.

Non-finite verbs are considered relatively easy structures to acquire and are marked similarly in the L1 and L2 of our participants. We therefore expect to see essentially native-like processing for violations in these constructions (similar to [30,31,33]), irrespective of the AoA of the participants. Yet, AoA effects may arise in the form of latency effects, which have sometimes been found in the few cases where they have been explicitly investigated [30]. While such latency effects are difficult to detect with traditional analyses which average across time-windows, the GAM approach has the potential to identify them.

In contrast to non-finite verbs, grammatical gender agreement is a notoriously difficult property for L2 learners. Previous research has shown that in some circumstances native-like processing for gender violations is possible, in some cases even if acquisition started later in life [29,46–50]. However, other studies indicate that with increased difficulty levels (e.g., in non-canonical word order; when L1 and L2 are less similar; with increased linear distance between agreeing elements; when the L2 gender system is non-transparent) learners resort to alternative processing strategies [29–31,46,47].

In the current study we test a group of learners for whom acquiring the L2 gender system poses a relatively difficult task. Their L1 and L2 are typologically distant and differ on many linguistic levels, the L1 being a Slavic and the L2 a Germanic language. Most interesting for the current study are the differences between the gender systems. Although German, Polish and Russian all have a three-way gender system consisting of masculine, feminine and neuter nouns, only Polish and Russian have additional subgenders based on animacy and/or personhood. More importantly, gender in Polish and Russian is overt and can be extracted from the phonological and morphological characteristics of the noun itself (i.e. in Polish masculine nouns generally end in consonants, feminine nouns in ‘-a’ and neuter nouns in ‘-o’ or ‘-e’; in Russian gender is determined by the declensional type of the noun: declinable nouns of declensional type I are masculine, nouns of declensional type II and III are feminine, while the rest are neuter [51]). In contrast, the gender system of German nouns is more complex and appears arbitrary and largely unpredictable. Furthermore, whereas in German the definite article most recognizably shows the gender of a noun, Polish and Russian have no articles (but do have other determiners, numerals, adjectives, pronouns and verbs that must agree in case, number and gender with the noun). The lack of articles in their L1 may make it more difficult for these learners to recognize incorrect gender marking on these elements in German, which is why we chose this particular construction for the materials of the present experiment. Based on these structural differences between the gender systems, we predict our group of learners to be particularly prone to AoA effects on gender processing.

We study the grammatical performance of our participants both by recording their brain responses to correct and incorrect use of the two types of constructions, and by registering their conscious perception of agreement violations in the form of end-of-sentence grammaticality judgments. In this way, the neural measurements can be compared to the outcome of processing. A divergence between these measures may come about due to behavioral task demands or the use of compensatory strategies [52].

In sum, the first goal of the present study is to investigate whether and in what sense the ERP brain response to L2 grammar processing is a function of AoA. To answer this question, we use a novel statistical approach that allows for a highly detailed analysis of the ERP signal. Our second goal is to establish in what sense AoA effects on L2 gender processing are dependent on the grammatical construction under investigation. Our third and final goal is to compare AoA effects on an online (unconscious) neural measure to a behavioral measure that reflects conscious grammatical processing.

## Materials and Methods

### Participants

101 participants took part in the experiment. Six participants had to be excluded from the analyses because of an exceedingly high number of artefacts in their EEG signal. Of the remaining participants, 66 were Slavic learners of German (15 Polish, 51 Russian), and 29 were native speakers of German. No differences between Polish and Russian learners were found in any of the behavioural measures described below (see Supporting Information: S2 File): logistic mixed-effects regression analyses on the gender assignment task and the grammaticality judgment task showed no L1 effect (*β* = -0.39, *SE* = 0.52, *p* = .450 and *β* = 0.31, *SE* = 0.35, *p* = .390, respectively) and neither did a linear regression analysis on the C-test (*β* = 1.55, *SE* = 3.42, *p* = .653). We therefore decided not to distinguish between Polish and Russian learners in the analyses reported in this paper. All participants were right handed, neurologically unimpaired and did not have any problems with hearing, speaking or writing.

The experimental procedures were approved by, and in accordance with the ethical guidelines of the Neuroimaging Center Groningen research ethics committee and the Ethikkommission des Instituts für Psychologie und Arbeitswissenschaft (IPA) of the Technical University of Berlin. All participants gave their written informed consent prior to participating in the study, they were notified that they were at liberty to withdraw from the study at any point and they received a small fee for their participation.

Participant characteristics and proficiency scores can be found in Table 1. All learners had moved to Germany between the ages of 7 and 36, and had been immersed in the L2 context for at least four years at the time of testing. About 40% of the learners had had some prior exposure to German in a classroom setting (during high school) in Russia or Poland. However, for the majority of individuals the age of first exposure was identical to the age of arrival in Germany. Given that the quality and quantity of input differs heavily between the classroom setting and an immersed context, exposure before arrival was disregarded and age of arrival in Germany was taken as the most representative measure of age of acquisition. Table 1 shows that the learner and native group are significantly different with respect to age at testing, with the native group being on average nine years older. Although increasing age can have a negative effect on language performance later in life, we do not expect substantial effects here since all participants are below the age of 60. Furthermore, if there were such an effect, it would not disadvantage the learners, since they are on average younger than the natives.

**Table 1:** Means (and ranges) of participant characteristics, scores on proficiency measures, and significance of between-group comparisons (Mann-Whitney U test).

Characteristic or measure		Learners (*n* = 66)	Natives (*n* = 29)	*U* and *p*-value
**Age and exposure:**	**Age at testing in years**	28.9 (18–53)	37.8 (22–58)	*U* = 498, *p* < .001
	**AoA in years^a^**	17.7 (7–36)	–	–
	**AoE in years^b^**	15 (7–32)	–	–
	**LoR in years^c^**	11.3 (4–25)	–	–
**Proficiency measures:**	**C-test in %^d^**	80.9 (51–95)	93.2 (86–98)	*U* = 198, *p* < .001
	**Gender assignment in %^e^**	93.3 (72.9–100)	99.9 (99–100)	*U* = 242, *p* < .001

^a^ Age of acquisition (= age of arrival in the L2 country)

^b^ Age of first exposure to the L2 (either in the L2 country or in a classroom setting outside of the L2 country)

^c^ Length of residence in the L2 country

^d^ Percentage of correct responses in the C-test

^e^ Percentage of correct responses in the pen-and-paper gender assignment task

A pre-selection on the basis of a condensed Goethe Institute placement test (a subset of items taken from http://www.goethe.de/lrn/prf/deindex.htm) ensured that all participants had a relatively high level of proficiency in German: participants had to complete at least 20 out of 30 items correctly to be selected for participation. Another proficiency measure was collected in the lab, in the form of a C-test (constructed by Schmid [53], available at www.let.rug.nl/languageattrition), which consisted of two texts containing gaps where alternating words missed half of their letters at the end. The participant’s task was to fill in these gaps. The total score was computed as the percentage of gaps that were filled in correctly (spelling errors were not penalized). After the EEG experiment, participants were also asked to complete an offline (pen-and-paper) gender assignment task. This task was used to test the participants’ knowledge of the grammatical gender of the critical target nouns used in the EEG experiment. The nouns were presented in randomized order and each item appeared three times. Participants were asked to indicate, by circling either the masculine (*der*) or neuter (*das*) definite article, whether they thought the noun was grammatically masculine or neuter in German (no feminine target nouns were included in the experiment, see below). The total score was computed as the percentage of items for which a minimum of 2/3 instances was assigned the correct gender. Participants’ scores on the proficiency measures can be found in Table 1.

To investigate the relation between AoA and the other participant characteristics, pairwise correlations between AoA, age at testing, length of residence, L2 proficiency (as measured by the C-test), and offline gender knowledge (as measured by the gender assignment task) were calculated. Because of skewed distributions, AoA, age at testing and length of residence were log-transformed, and C-test and gender assignment task scores were arcsine transformed. False discovery rate correction [54] was applied to the *p*-values to account for multiple comparisons. We found a relatively strong positive correlation between AoA and age at testing (*r*(64) = 0.71, *p* < .001). We decided to include AoA, and not age at testing, as the relevant predictor in the analyses reported in this paper. Although we have good reasons to assume the effects we report can be attributed to AoA (see the Introduction of this paper), we cannot rule out the possibility that (part of) the effects might be due to age at testing, as these factors are inevitably confounded to the extent that it is impossible to tease them apart [55]. All other variables only correlated moderately with AoA in the expected direction: lower AoA is associated with longer length of residence (*r*(64) = -0.45, *p* < .001), higher L2 proficiency (*r*(64) = -0.43, *p* < .001), and better gender knowledge (*r*(64) = -0.56, *p* < .001).

### Materials

The design of the EEG experiment was largely based on work by Loerts [33], who studied L2 gender and non-finite verb processing in native Dutch speakers and Polish learners of Dutch. The present study was part of a larger research project on L2 acquisition and L1 attrition. We confine us to the materials and methods of the current sub-project below. We refer the reader to [56] for an in-depth description and detailed discussion of the research design of the full project.

For the current experiment, 144 German experimental sentences were created (see Table 2 for examples, the full list of sentences can be found in the Supporting Information: S1 Table). To reduce fatigue effects, we used a limited number of 48 sentences (i.e. 24 grammatical and 24 ungrammatical trials per subject) for the highly salient verb agreement violations, which have been shown to elicit large ERP effects. In the less salient gender condition, however, we increased the statistical power by including twice as many sentences. There were no sentences that contained ungrammaticalities in both verb and gender agreement.

**Table 2:** Example materials of the EEG experiment.

Condition	Example sentences
**Verb agreement**	Leider hat die Rose diesen Herbst noch nicht geblüht/*blühen, obwohl wir sie jeden Tag gegossen haben.
	Unfortunately this fall the Rose has not yet bloomed/*bloom, *although we watered it every day*.
	Während der Zirkusvorstellung mußten die Kinder die ganze Zeit lachen/*gelacht, da die Clowns so witzig waren.
	During the circus show the Kids had to laugh/*laughed the entire time, *because the clowns were so funny*.
**Gender agreement**	Nach der Schlägerei ist das/*der Auge des Angestellten von der Krankenschwester versorgt worden.
	*After the fight the*_*neut*_ /*the_*masc*_ *eye of the worker was treated by the nurse*.
	Glücklicherweise ist der/*das frühe Beginn der Konferenz nach hinten verschoben worden.
	Fortunately the_masc_/*the_neut_ early beginning of the conference was postponed.

The critical targets where the ERP was measured are underlined and the * indicates the incorrect verb form or determiner.

Half of the 48 sentences used to test non-finite verb agreement contained an infinitive and the other half contained a past participle. For their ungrammatical counterparts, these verbs were altered into their participial or infinitival form, respectively. Infinitive vs. past participle construction was not included as a factor, but merely served to decrease the predictability of the violation.

In the 96 sentences that were used to test grammatical gender agreement the critical noun phrases were either masculine and neuter. Feminine forms were avoided since the feminine singular article overlaps with the general article for plural nouns of all genders. Agreement was manipulated via the definite article, which either agreed in gender with the succeeding noun or violated gender concord. Article and noun were either adjacent or non-adjacent (with an adjective intervening between the article and noun), again to decrease the predictability of the violation. Note that in German an adjective following a definite article is never gender marked, hence all violations occurred on the noun. All critical noun phrases were in the nominative case, and, to avoid effects of semantic gender, all target nouns referred to inanimate entities. The onset of the sentences (the region before the critical noun phrase) was varied using adverbs, complementizer phrases and prepositional phrases; the number of these initial structures was balanced across conditions. Only highly frequent critical nouns and verbs were used (nouns: mean 1.62, range 0.38–2.70; verbs: mean 1.78, range 0.29–2.86, on log lemma frequency of occurrence per million taken from the *Deutsche Referenzkorpus*, [57]).

Since both the learners' mother languages encode gender, effects of cross-linguistic similarity are a potential confound. However, in the current study we did not expect to find a significant effect of cross-linguistic similarity (between L1 and L2) of the gender of the target nouns, for several reasons. First, linguistic distance between Polish/Russian versus German is large, while cross-language gender compatibility effects are usually found in languages more closely related (e.g. [58]). Second, there is no one-to-one correspondence between the L1 and L2 gender systems of the participants (as was described in the introduction), making transfer effects less likely. Finally, we did not expect transfer effects to play a large role in our participants, since the learners are at the end stage of learning, not at the beginning of the acquisition process. Their high accuracy scores on the gender assignment task (93% on average, Table 1) show that they have learned the correct gender of most of the nouns. We ran an analysis on the gender assignment task and the grammaticality judgement task to investigate whether participants were more accurate when nouns had the same gender in their two languages (see Supporting Information: S2 File). We found no effect of gender similarity in the grammaticality judgment task (*β* = 0.12, *SE* = 0.07, *p* = .092), while there did appear to be a significant effect in the gender assignment task (*β* = 0.90, *SE* = 0.24, *p* < .001). However, effect size is very small. The model predicts a probability of 0.967 for a correct answer when the gender of a noun is different between L1 and L2, and a probability of 0.986 when the gender is the same. This difference is so small that we decided not to take this factor into account in the analysis reported in this paper. While effects of cross-linguistic similarity are an interesting topic that deserves further research, we will not focus on them here.

In addition to the experimental items, a list of 134 well-formed filler sentences was included. These filler sentences were created such that they balance the number of active, passive, one-clause and two-clause sentences, in order to reduce pattern learning and strategic processing in participants. Furthermore, they lowered the overall proportion of ungrammatical sentences to around a quarter, making the task slightly more similar to natural language processing.

Spoken forms of all sentences were recorded. Each sentence was read aloud by a female native speaker with a standard German accent who was trained to produce grammatical and ungrammatical sentences with normal intonation. In half of the sentences, target regions and preceding context were cross-spliced from correct to incorrect sentences and vice versa to avoid potential confounds in the form of prosodic cues. Noise reduction and volume normalization were applied to all sound files. Four experimental lists were created using a Latin Square design, crossing the factors grammaticality (grammatical, ungrammatical) and splicing (spliced, unspliced), to ensure each participant was presented with only one version of each sentence and an equal number of each type. Each list was presented to an approximately (due to randomization) equal number of participants and each participant was only exposed to a single list.

### Procedure

The EEG was recorded while participants listened to the sentences. After each sentence, participants made a grammaticality judgment by means of pressing one of two buttons. The sentences were presented at normal speech rate, using E-prime [59,60] and a set of audio speakers that were placed to the left and right side of a computer screen. The grammaticality judgments were recorded in E-prime. The visual stimuli (a fixation cross during sentence presentation and the grammaticality judgment question) were presented on the screen in front of the participants. During sentence presentation, participants were instructed to avoid moving any parts of their body, including eye movements and blinking. The stimuli were presented in four blocks, and the duration of the breaks between these blocks was determined by the participants. Altogether, this task lasted about one hour. Subsequently, participants were asked to fill in the pen-and-paper gender assignment task. Here, all critical target nouns were presented three times, to minimize effects of guessing. The C-test was administered during another visit, which took place on a separate day (before the EEG experiment).

### EEG data recording and preprocessing

**Fig 4:**
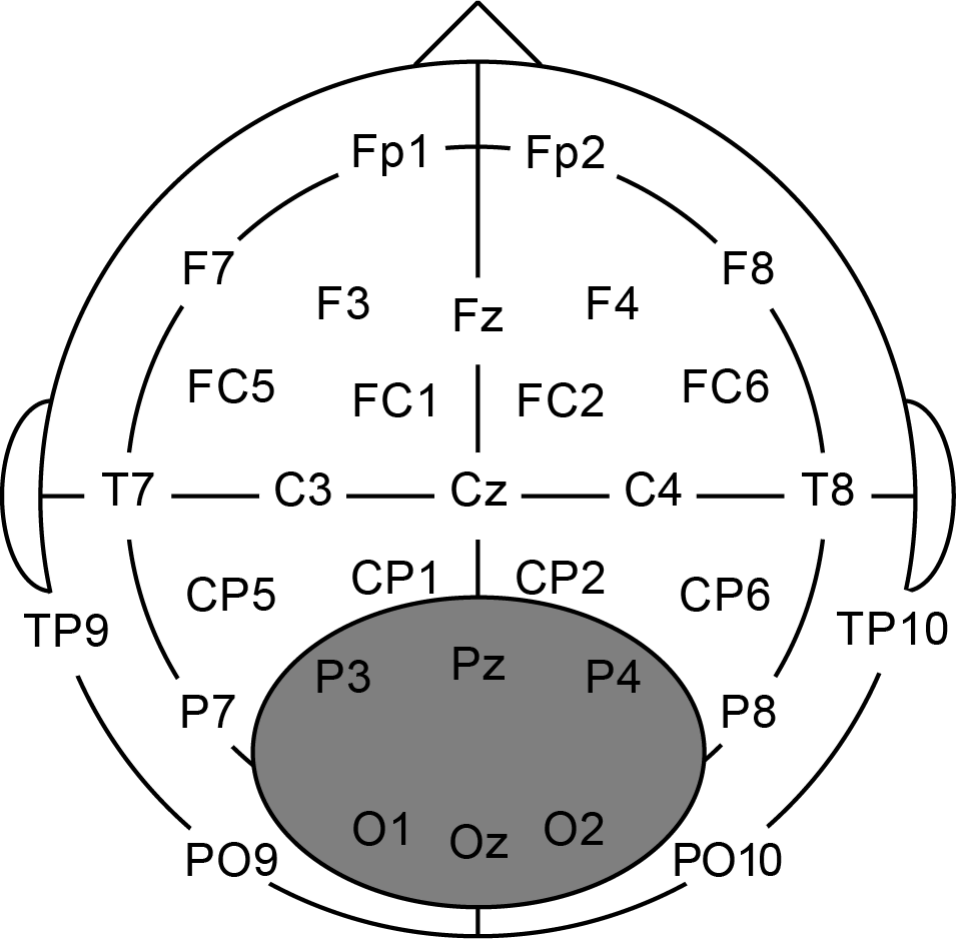
Location of the electrodes and ROI. Approximate location of the EEG recording sites and the central-posterior region of interest used for analysis.

Participants were tested at labs in three different German cities: Hamburg (*N* = 61), Berlin (*N* = 10) and Mainz (*N* = 24). For all locations, the continuous EEG (500 Hz/22 bit sampling rate) was recorded for 32 Ag/AgCl active (Berlin, Hamburg) or passive (Mainz) scalp electrodes (Brain Products) mounted in an elastic cap (Electro Cap International Inc.) according to the international 10–20 system (see Fig 4 for the locations of the electrodes). To monitor horizontal eye movement, electrodes were placed at the outer canthi of each eye. Vertical eye movement was recorded either using electrodes placed above and below the right eye (Berlin, Mainz), or two electrodes attached below both eyes, for which Fp1 and Fp2 served as a reference (Hamburg). All signals were measured against either a nose reference (Berlin, Hamburg) or a right-mastoid reference (Mainz) and an electrode placed on the left cheek (Berlin, Hamburg) or the sternum (Mainz) served as ground.

After acquisition, the raw data were further processed with Brain Vision Analyzer 2.0.4. The data were re-referenced to the average of two electrodes placed over the left and right mastoids (TP9 and TP10) and digitally filtered above 0.1Hz and below 40Hz. The data were segmented, time-locked to the onset of the critical target and a baseline period was set from 200 ms to 0 ms before onset of the target to normalize the data. If there were ocular artefacts, these were corrected [61]. Average ERPs were formed without regard to behavioral responses, from trials free of artefacts (individual channel artifacts led to rejection of 2.2% of the data in the learner group and 0.6% in the native group). A central-posterior region of interest (ROI) containing six electrodes was used for analyses (depicted in Fig 4), consistent with the location in which other studies on grammatical gender processing have found the (late stage) P600 effect to be most pronounced (see, e.g., gender studies in [20]). Clearly, the selection of a specific ROI does add some subjectivity to our analysis. However, even though it is possible to take into account multiple electrodes/ROIs in a GAM analysis, at present the high computational requirements make this additional complexity infeasible. Finally, the data were down-sampled to 100 Hz for analysis (to reduce processing time of the GAM model).

### Analyses

All analyses were performed in R (version 3.1.2: [62]); specific packages used are mentioned below. The data and commands used for the analysis are available in the Supporting Information: S1 File (data) and S2 File (analysis). For reproducibility, the data, analysis and results are also available as a paper package stored at the Mind Research Repository (http://openscience.uni-leipzig.de).

#### Logistic mixed-effects regression analysis of the behavioral data

We analyzed the accuracy of the answers to the grammaticality judgments by fitting a mixed-effects logistic regression model, using the lme4 package (version 1.1.7: [63]). The dependent variable was accuracy of the answer (correct: 1 or incorrect: 0), and we assessed the importance of *group* (natives vs. learners), *structure* (verb vs. gender) and *grammaticality* (grammatical vs. ungrammatical) as predictors. Additionally, we investigated the effect of *AoA* as a predictor in the learner group only (the intercept was removed from this model, in order to estimate separate intercepts for each level of the combination of predictors). We included the maximal random effect structure supported by the data. Trials for which no response was registered were removed from the data before analysis (2.4% data loss).

#### ANOVA and correlational analysis of the EEG data

To highlight the differences between the GAM analysis and the traditional ANOVA analysis, we first analyzed average amplitudes of the ERP waveforms in the time-window in which a P600 is to be expected: 500–1200 ms after stimulus onset. For the grand mean analysis an ANOVA was performed using the ez R package (version 4.2.2: [64]), by calculating group averages over subjects and items, dividing the learners into early (AoA 7–16, *n* = 26) and late (AoA 17–36, *n* = 40) learners. A Levene's test showed that the variances were equal across the groups (see Supporting Information: S2 File). Note that the group split has been made at an arbitrary point (at about the end of puberty). We want to emphasize that the experiment was set up to treat AoA as a continuous predictor, as we did in the GAM analysis (see below). In fact, we recommend *against* using ANOVAs for this particular research question, one of the clear drawbacks being the necessity to create this arbitrary group division.

*Group* (natives vs. early learners vs. late learners), *structure* (verb vs. gender) and *grammaticality* (grammatical vs. ungrammatical), were included as factors in the ANOVA. Only main effects of, and interactions with, grammaticality were interpreted, since our interest lies in the differences between amplitudes for grammatical and ungrammatical sentences. False discovery rate correction [54] was applied to the *p*-values for follow-up tests to control for Type 1 errors. In the presence of a significant higher-level interaction, lower-level interactions and main effects are not interpreted.

Additionally, we performed a correlational analysis on the full AoA range across both learner groups. In pairwise correlations we tested the relation between AoA and the average magnitude of the difference wave (ungrammatical minus grammatical) in the 500–1200 ms time window, in the verb and the gender condition separately.

#### GAM analysis of the EEG data

GAM analyses were performed using the mgcv R package (version 1.8.4: [65,45]) and results were plotted using the itsadug R package (version 1.0.1; [66]). We used GAMs to assess the (possibly non-linear) effect of *AoA* on the non-linear ERP signal over *time*. We included the time-locked EEG signal per trial (i.e. per sentence per participant) of the learner data, in the full time range of -500 to 1400 ms before/after target onset. *Grammaticality* was converted into a binary variable (ungrammatical: 1 vs. grammatical: 0), to explicitly test for the non-linear difference between the two levels of grammaticality (i.e. to model the difference wave). To assess if a non-linear interaction between time and AoA was necessary, we fitted a model that investigated the independent contribution of AoA vs. time on this difference, by means of decomposing the main effects of each of these factors and their pure interaction. The model also included the time trends of individual participants per structure and level of grammaticality, and the time trends of individual items per level of grammaticality, to account for participant and item variation. Furthermore, each model included a parameter to correct for the presence of autocorrelation in the residuals.

As the response variable in our models (i.e. the ERP signal) was heavy-tailed, causing the residuals of our model to be non-normal, we fitted the generalized additive model using the scaled-t family. As a consequence, the residuals followed the standard normal distribution.

Since a GAM is a regression model, it provides the additional possibility to include potential nuisance factors. Consequently, we included two potential sources of variability between subjects and items: test location and word frequency. Effects of test location are a potential source of concern in a study for which data are collected at various locations [56], and should therefore always be investigated. Item variation is hardly ever dealt with in traditional analysis, but especially in L2 learners it is conceivable that word frequency may affect ERP responses.

## Results

### Behavioral results for the grammaticality judgment task

**Fig 5:**
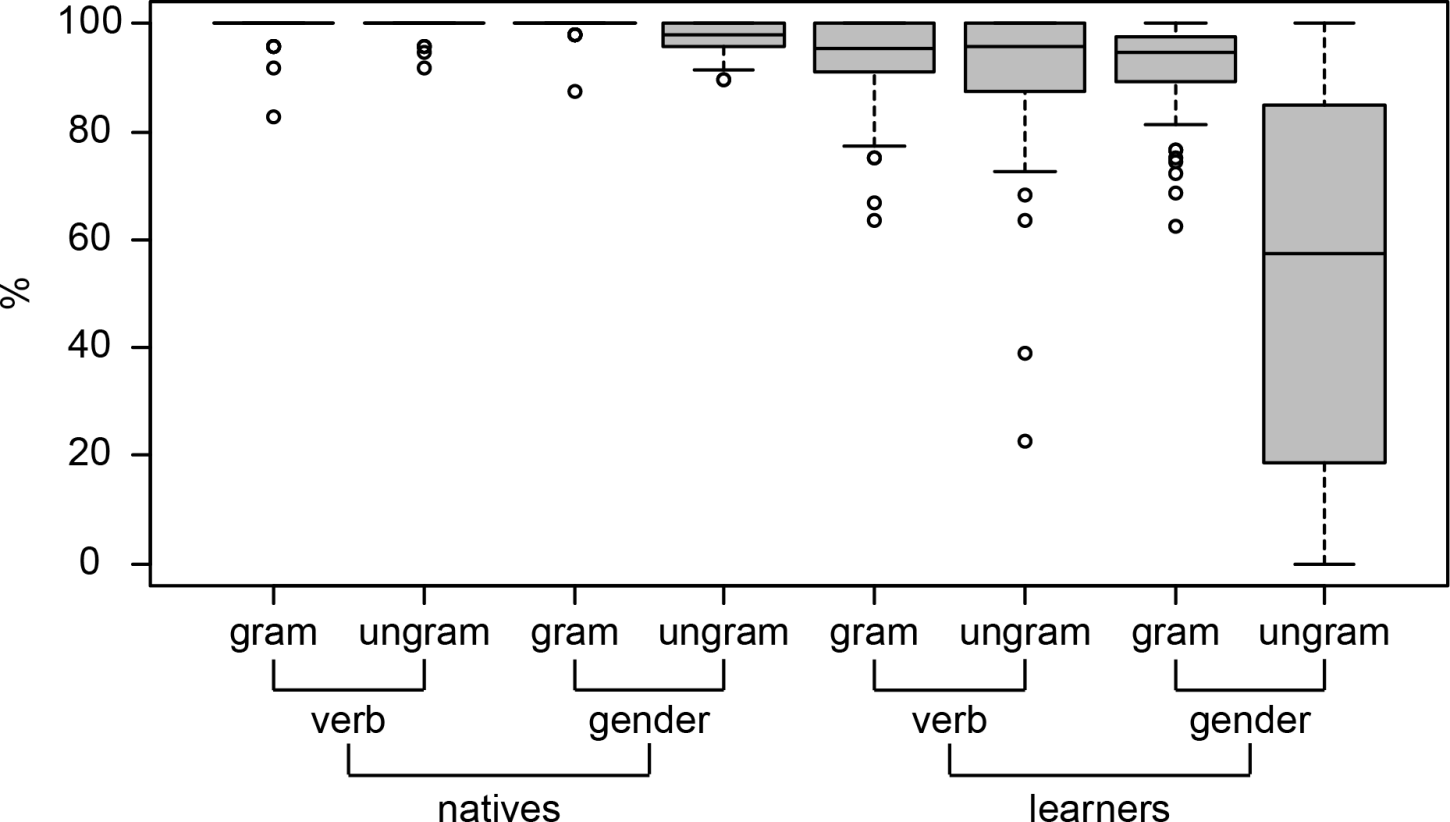
Accuracy on (behavioral) grammaticality judgments made during the EEG recording session. Performance in percentage of accurate responses is plotted separately for each level of group (learners, natives), structure (verb, gender) and grammaticality (grammatical, ungrammatical).

The distribution of the percentage of accurate grammaticality judgments across participants is shown in Fig 5. As is to be expected from this figure, a mixed-effects logistic regression model revealed significantly lower proportions of accurate responses in learners as compared to natives, and significantly lower accuracy scores in the ungrammatical compared to grammatical sentences for both groups in the gender condition (the results of this model are shown in the Supporting Information: S2 File).

Table 3 shows the results of another model, which investigated the AoA effect in each of the conditions. We observe significant AoA effects in all four conditions, with the smallest adjustment for grammatical verb sentences (*β* = -0.09, *SE* = 0.02, *p* < .001) and the largest for ungrammatical gender sentences (*β* = -0.20, *SE* = 0.01, *p* < .001). Using the inverse logit transformation, we can calculate that for grammatical verb sentences a learner with an AoA of 7 has a probability of 0.98 for an accurate answer. That probability decreases to 0.80 for a learner with an AoA of 36. For ungrammatical gender sentences the corresponding probabilities vary from 0.92 to 0.03.

**Table 3:** Results summary of the mixed-effects logistic regression analysis of the AoA effect in the grammaticality judgments task.

Predictor				
*Random effects*:	**Variance**			
Item (intercept)	0.15			
Subject (intercept)	0.96			
*Fixed effects*:	**Estimate**	**Std. error**	***z*-value**	***p*-value**
Struc: verb × Gram: grammatical	4.59	0.30	15.49	< .001
Struc: gender × Gram: grammatical	4.87	0.26	18.76	< .001
Struc: verb × Gram: ungrammatical	6.19	0.39	15.94	< .001
Struc: gender × Gram: ungrammatical	3.96	0.22	18.16	< .001
Group: learners × AoA × Struc: verb × Gram: grammatical	-0.09	0.02	-5.52	< .001
Group: learners × AoA × Struc: gender × Gram: grammatical	-0.11	0.01	-7.59	< .001
Group: learners × AoA × Struc: verb × Gram: ungrammatical	-0.18	0.02	-9.28	< .001
Group: learners × AoA × Struc: gender × Gram: ungrammatical	-0.20	0.01	-16.14	< .001

Predictors are abbreviated (Struc = Structure, Gram = Grammaticality, Group = Group) and followed by the relevant level. A positive estimate indicates that the value for this predictor increases the proportion of correct responses, while a negative estimate indicates the opposite effect.

### EEG results: traditional analysis (ANOVA and correlation)

To illustrate the potential benefits of using GAMs compared to a more traditional approach, we first present an ANOVA and a correlational analysis of the ERP data. In the next section, a GAM analysis of the same dataset will be presented.

**Fig 6:**
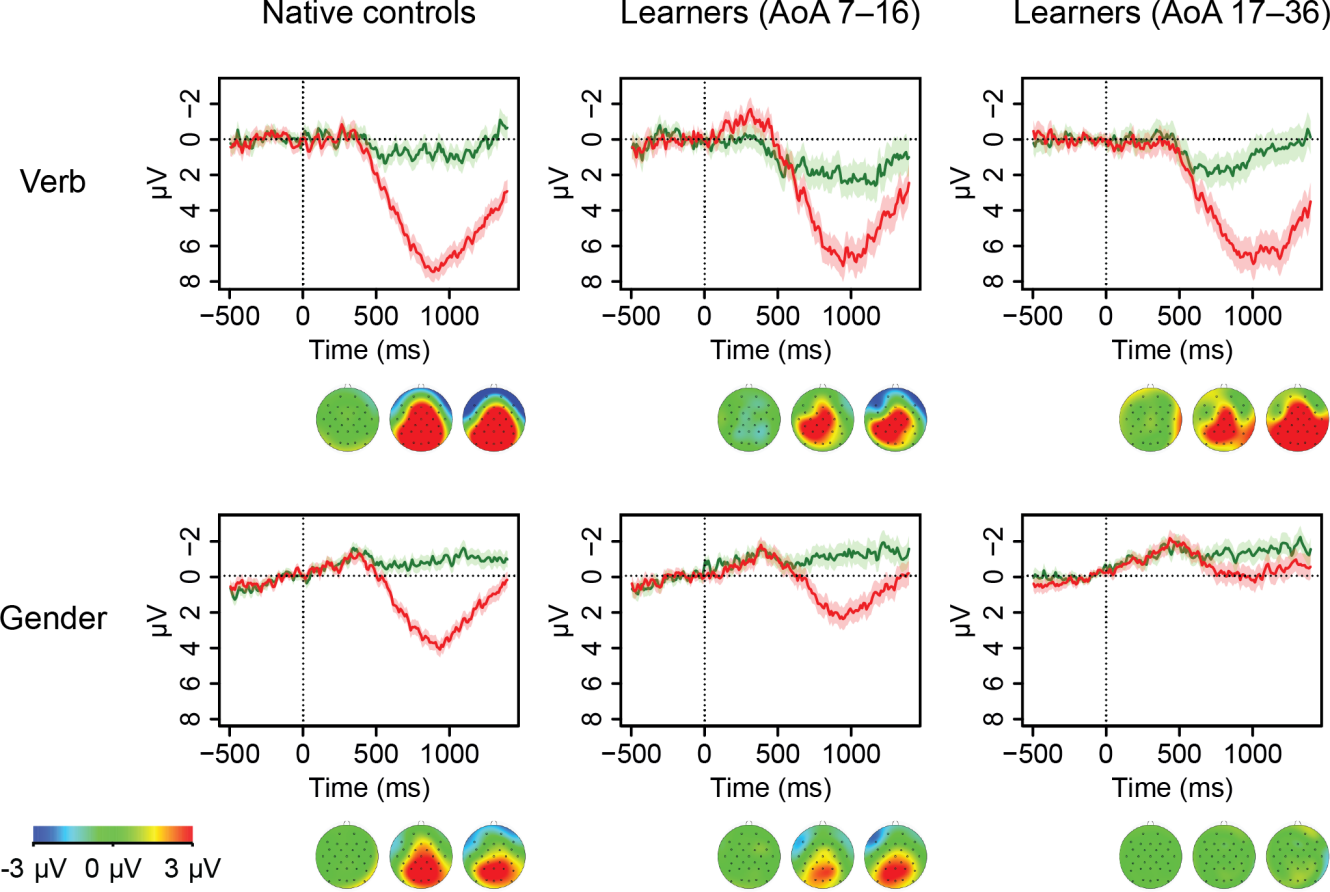
Grand average ERP responses. Waveforms show the ERP signal averaged over the electrodes in the central-posterior region of interest (see Fig 4) for natives and learners. The green lines represent the average response to grammatical targets, the red lines show the response to violations. Underneath the waveform graphs, voltage maps are plotted, which show the scalp topography of the effect in the difference wave (i.e. mean amplitude in ungrammatical minus grammatical condition) in the 0–500, 500–1000 and 1000–1400 ms time window. The scalp topographies are visualized looking down on the head (i.e. the location of the nose is at the top), with positive differences in red, negative differences in blue, and green representing no difference. A traditional group split divides learners into early (7–16) and late (17–36) age of onset of acquisition groups. Both natives and learners show P600 effects for non-finite verb agreement. Only early learners show the same effect for gender agreement, whereas late learners do not show sensitivity for gender violations.

Fig 6 shows the grand average ERP waveforms for the central-posterior region (see Fig 4 above), for natives and learners in each of the conditions. Additionally, the scalp distribution of the effects is shown in voltage maps. A group split was used to obtain an indication of the effect of AoA in the learner group: the waveforms for early learners (AoA 7–16, *n* = 26) are plotted separately from the ones of the late learners (AoA 17–36, *n* = 40). Note that the cut-off point for this split is arbitrary (at about the end of puberty). The first row of Fig 6 shows that early as well as late learners show a P600 effect similar to natives for non-finite verb agreement. The second row shows that for gender agreement, early learners show a P600 effect similar to native speakers. In contrast, late learners do not show sensitivity to gender violations.

These observations are supported by an ANOVA on the average amplitudes in the 500–1200 milliseconds time window, revealing significant interactions between group and grammaticality (*F*(2,92) = 4.23, *p* = .017) and between structure and grammaticality (*F*(1,92) = 10.33, *p* = .002). Follow-up analyses conducted separately per group revealed that only in late learners and natives (and not in early learners) there is a significant structure by grammaticality interaction (*F*(1,39) = 11.99, *p* = .004; *F*(1,28) = 7.09, *p* = .019, and *F*(1,25) = 0.15, *p* = .702, respectively). Follow-up analyses per structure in these groups revealed significant differences between grammatical and ungrammatical sentences in both groups and structures, except for the gender condition in late learners (natives: verb *t*(50.44) = -6.08, *p* < .001, gender *t*(47.35) = -7.39, *p* < .001; late learners: verb *t*(67.90) = -4.34, *p* < .001, gender *t*(70.94) = -1.16, *p* = .251).

**Fig 7:**
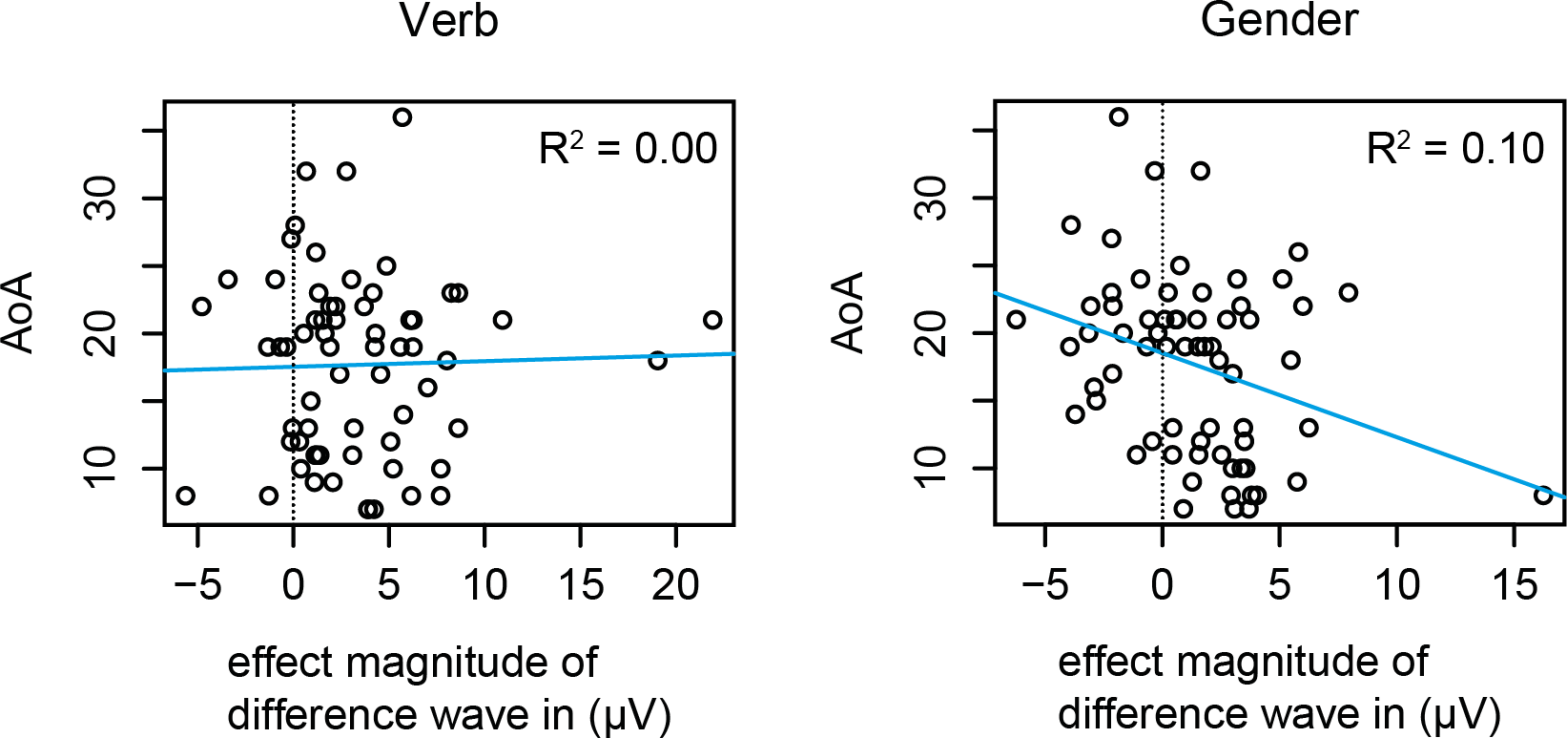
The effect of AoA on the average ERP signal. The figure shows the average magnitude of the difference wave per individual (*x*-axis), against that individual’s AoA (*y*-axis). This magnitude was calculated by taking the ERP signal per condition, averaged over the electrodes in the central-posterior region of interest (see Fig 4) using the mean amplitude in the 500–1200 ms time window, to calculate the difference between the individual’s response to ungrammatical versus grammatical targets. The plots reveal a weak correlation between AoA and the magnitude of the grammaticality effect in the gender condition (*r*(64) = -0.32, *p* = .009), but no effect in the verb condition (*r*(64) = 0.03, *p* = .825).

In order to investigate the strength of the relation between AoA and the P600 effect, we performed a correlational analysis on the basis of individual amplitudes in both learner groups taken together. Fig 7 shows the average magnitude of the difference wave (ungrammatical minus grammatical) in the 500–1200 ms time window per individual. We see a weak AoA effect in the gender condition (*r*(64) = -0.32, *p* = .009), but no effect in the verb condition (*r*(64) = 0.03, *p* = .825). However, this analysis is based on averages (over time and over items), and therefore the details of the waveforms are lost. To take into account the full range of variability in AoA as well as over time and across items, we present the GAM analysis of the data in the next section.

### EEG results: GAM analysis

Table 4 summarizes the results of the GAM model investigating the effects of time and AoA on the difference wave for verb and gender sentences. We want to note that in a separate model (results available in the Supporting Information: S2 File) we have tested whether the effect of a gender violation is significantly different from the effect of a verb violation. This was the case for both the main effect of time and the interaction between time and AoA, but not for the main effect of AoA. The differences between the structures justify that we investigate them separately, each represented by their own factor.

**Table 4:** Summary of the results of the GAM model for the effects of time and AoA, for verb and gender violations.

	***Parametric coefficients*:**	**Estimate**	**Std.error**	***t*-value**	**Pr(>|t|)**	
1	(Intercept)	0.91	0.29	3.14	0.002	**
2	Structure: gender	-1.38	0.39	-3.58	< 0.001	***
3	VerbUngrammatical	1.50	0.41	3.66	< 0.001	***
4	GenderUngrammatical	0.49	0.36	1.34	0.180	
	***Smooth terms*:**	**Edf**	**Ref.df**	**F**	***p*-value**	
5	s(Time), Structure: verb	6.59	7.67	12.83	< 0.001	***
6	s(Time), Structure: gender	2.24	2.71	16.53	< 0.001	***
7	s(AoA), Structure: verb	1.01	1.01	0.37	0.545	
8	s(AoA), Structure: gender	1.29	1.30	0.47	0.544	
9	ti(Time, AoA), Structure: verb	2.05	2.27	1.94	0.135	
10	ti(Time, AoA), Structure: gender	1.97	2.12	1.79	0.164	
11	s(Time), VerbUngrammatical	7.77	8.51	37.64	< 0.001	***
12	s(AoA), VerbUngrammatical	1.01	1.01	0.29	0.592	
13	ti(Time, AoA), VerbUngrammatical	3.88	3.96	10.24	< 0.001	***
14	s(Time), GenderUngrammatical	7.79	8.60	18.29	< 0.001	***
15	s(AoA), GenderUngrammatical	1.06	1.06	2.19	0.135	
16	ti(Time, AoA), GenderUngrammatical	5.69	6.86	5.65	< 0.001	***
17	s(Time, SubjectGramStruc)	607.83	2368.00	1.90	< 0.001	***
18	s(Time, WordGram)	250.23	2588.00	0.84	< 0.001	***

Since grammaticality was converted into a binary variable, ‘Ungrammatical’ models the difference wave. A positive estimate indicates that this predictor increases amplitudes, while a negative estimate indicates the opposite effect. The edfs (estimated degrees of freedom for the model parameter: the higher the more complex) provide an estimation of the complexity of the 1-dimensional smooth (s) or 2-dimensional surface (ti). Asterisks indicates significance of

* *p* < .05

** *p* < .01

*** *p* < .001. The explained deviance of this model is 3.27%.

Table 4 reports the results for the following model specification:

uV ~ s(Time, by = Structure) + s(AoA, by = Structure) + ti(Time, AoA, by = Structure) + s(Time, by = VerbUngrammatical) + s(AoA, by = VerbUngrammatical) + ti(Time, AoA, by = VerbUngrammatical) + s(Time, by = GenderUngrammatical) + s(AoA, by = GenderUngrammatical) + ti(Time, AoA, by = GenderUngrammatical) + Structure + VerbUngrammatical + GenderUngrammatical + s(Time, SubjectGramStruc, bs = "fs", m = 1) + s(Time, WordGram, bs = "fs", m = 1)

This specification indicates that our dependent variable is the ERP amplitude (in microvolts) which we are modeling by allowing a non-linear effect of time, AoA, and the non-linear interaction between the two (i.e. s(Time), s(AoA), and ti(Time, AoA), respectively). Essentially, we are fitting these effects for two structures and for both grammatical and ungrammatical targets (i.e. four combinations). Rather than obtaining separate patters for each combination (e.g., grammatical verb), we specify the model in such a way that we directly obtain the difference between ungrammatical and grammatical targets (which models the difference wave, i.e. the P600 in this case). Consequently, the first three items of the model specification fit the non-linear pattern over time, AoA, and the non-linear interaction between time and AoA for the grammatical targets (separately for verb and gender, due to the ‘by = Structure’ designation), whereas the next six items correspond to the difference between the ungrammatical and the grammatical targets for each of the three types of patterns (over time, over AoA, and the interaction between time and AoA) and the two structures. As all non-linear patterns are centered, the inclusion of the fixed-effect predictors ‘Structure’, ‘VerbUngrammatical’ and ‘GenderUngrammatical’ is necessary to model constant differences between these levels. The final two items in the model specification represent the random effects and model the individual (non-linear) variability in the EEG signal (per subject, separately for the four combinations of structure and grammaticality, and per item, separately for its grammatical and ungrammatical version).

The results of the model fit are shown in rows 1 to 18 of Table 4. In rows 1 to 4 we see the estimates for the parametric coefficients. These show that gender targets overall have more negative amplitudes, ungrammatical verb targets generally give rise to more positive amplitudes compared to grammatical verb targets, and ungrammatical gender targets are not significantly different from grammatical gender targets. However, this parametric part of the model is not very informative, as it does not distinguish between different AoA or time values. Below Table 4 it is reported that the deviance explained by the model is 3.27%. This low value is due to the large amount of noise and great deal of individual variability that is common to EEG data.

Rows 5 to 10 show the significance of the non-linear patterns associated with grammatical verb and gender items (see explanation of the model specification above). However, as we are interested in the difference between ungrammatical and grammatical items, we focus on rows 11 to 16. These provide information (separately for verb and gender) about the individual contribution of time and AoA to this difference, and show whether a non-linear interaction between these factors is necessary. Significance of the non-linear patterns shown in these rows directly indicates significance of the ERP difference we are interested in.

**Fig 8:**
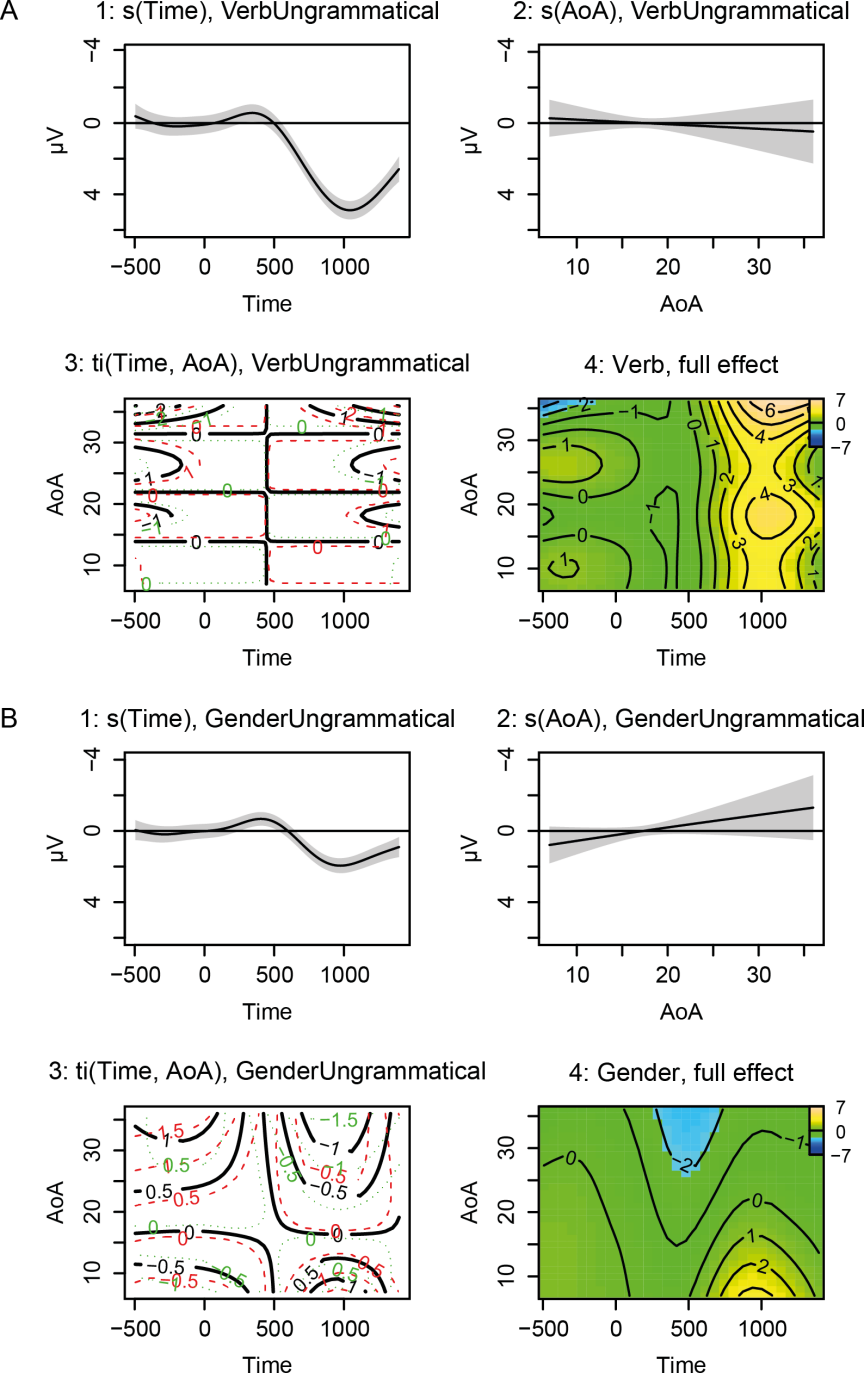
Visualization of the GAM model for the effects of time and AoA on the difference wave, for the verb (Panel A) and gender (Panel B) condition. First, the individual smooths and surfaces for the *additive* effects of time (Panels A1 and B1), AoA (Panels A2 and B2) and their pure interaction (Panels A3 and B3) are plotted separately. These are followed by a plot that summarizes all these effects together (the ‘full effect’ plots, Panels A4 and B4). After taking out the main effect of time, the effect of AoA is not significant on its own, for either verb or gender, but only in interaction with time (see Table 4). These adjustments to the general time pattern match the complexity of the AoA effects on timing, slope and polarity of the ERP components seen in the ‘full effect’ plots: In the verb condition, we see a P600 effect across all AoAs, with only minor adjustments to amplitude, onset and duration of this component for earlier vs. later learners. For gender, there is a diminishing P600 effect for early AoAs and instead a negativity that peaks around 500 ms for later AoAs.

Rows 11 and 14 show that the difference over time (i.e. the effect of an ungrammaticality) is significant for both verb (*F* = 37.64, *p* < .001) and gender constructions (*F* = 18.29, *p* < .001). The edfs (estimated degrees of freedom) provide an estimation of the complexity of the smooth (the higher, the more complex), showing that the time effect is highly non-linear in both conditions (both edfs > 7). These parameters should be interpreted along with their visualization, shown in Fig 8: Panels A1 and B1 show the smooth over time for verb and gender violations, respectively. Time in milliseconds is plotted against amplitude of the difference wave in microvolts. The general effect of time shows a P600 effect for both structures, with just a hint of a preceding negativity. The P600 peaks around 1000 ms after target onset, and is smaller in magnitude for gender compared to verb violations (the aforementioned model comparing both structures directly, available in the Supporting Information: S2 File, reveals this difference as significant).

As can be seen in Table 4 (rows 12 and 15), the main smooth for the effect of AoA is not significant for either verb (*F* = 0.29, *p* = .592) or gender violations (*F* = 2.19, *p* = .135). Both AoA effects are basically linear (edfs close to 1), but do not affect the general time pattern significantly. The accompanying plots in Fig 8 (Panels A2 and B2), in which AoA in years is plotted against amplitude of the difference wave in microvolts, illustrate the linearity and non-significance (i.e. confidence bands contain 0 over the whole timespan) of the AoA smooths.

Nonetheless, Table 4 also shows (rows 13 and 16) that the effect of AoA does reach significance in interaction with time: for both verbs (*F* = 10.24, *p* < .001; edf = 3.88) and gender (*F* = 5.65, *p* < .001; edf = 5.69) we see a significant non-linear interaction between these factors. In Fig 8, this interaction is plotted in Panels A3 and B3. Time in milliseconds (*x*-axis) is plotted against AoA in years (*y*-axis), while the third dimension (i.e. the contour lines) represents amplitude in microvolts. The contour lines connect points (i.e. combinations between time and AoA) of similar microvoltages. Confidence intervals for the contour lines are shown in green (lower bound) and red (upper bound). This complex surface shows the additive effect of the interactions relative to the overall effect of time (and to the non-significant overall effect of AoA). For verb violations, we see that AoA adjustments to the main time curve are mainly present at the very beginning of the time interval we are investigating (in which there are some minor effects in the baseline), versus the very end (in which the late positivity changes shape somewhat across different AoAs, i.e. steeper slope and sharper peak for earlier learners). For gender violations, we see AoA adjustments of the general time pattern starting slightly after 500 ms for the earlier learners (in which there is a P600), while for later learners the AoA adjustments (a decrease of the amplitudes) start somewhat earlier in time.

Since it can be challenging to read these plots and to imagine what these three additive effects (the smooths of time and AoA and the tensor for the interaction) together look like, Panels A4 and B4 of Fig 8 show a plot in which the full pattern (i.e. the three additive effects combined) is visualized. The *x*-axis represents time (in milliseconds) and the *y*-axis represents AoA values. The dependent variable (scalp voltage in microvolts) is represented by both black contour lines and colors. The colors follow the terrain color-scheme: a blue color (sea) represents low values, whereas the values increase going to green (plains) and finally yellow (slope of a mountain) for the highest microvoltages. The black contour lines again connect points (i.e. combinations between time and AoA) of similar microvoltages. The closer the contour lines are together, the steeper the slope.

The significant non-linear interactions we discussed earlier match the complexity of the patterns we see in these ‘full effect’ plots. For verb violations (Panel A4), we see a P600 effect across all AoAs. The graphs shows that the slope of the peak of the P600 has a slightly earlier onset and later offset as we get to higher AoAs (even beyond the end of the timespan of up to 1400 ms after target onset), and that it reaches a higher maximum.

For gender violations, the ‘full effect’ plot of Fig 8, Panel B4, shows a decreasing relationship between AoA and the size of the P600 effect: Learners having an AoA between 7 and about 20 are showing (diminishing) P600 effects in response to gender violations, but learners with later AoAs (ranging between about 25 and 36) instead show a posterior negativity. Furthermore, we notice some latency effects: The peak of the P600 becomes somewhat later the higher the AoA and the negativity for later learners starts (and peaks) somewhat earlier than the P600 for earlier learners.

**Fig 9:**
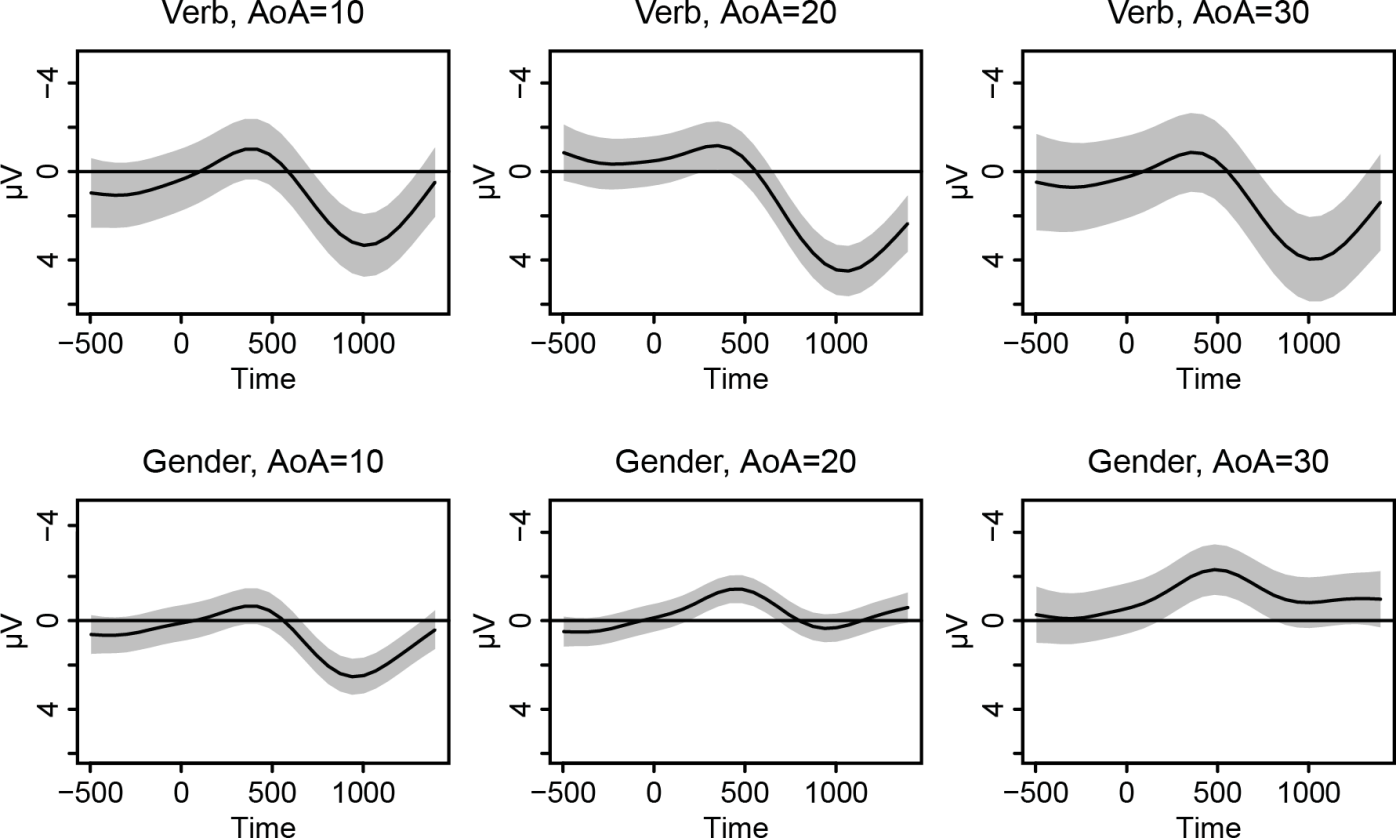
The effect over time on the ERP signal for learners with an AoA of 10, 20 and 30 for verb and gender violations. These plots present an alternative visualization of the two-dimensional ‘full effect’ surfaces of Fig 8: AoA values, which were represented on the y-axis in Fig 8 Panels A4 and B4, are now represented by three separate plots for particular AoA values, with microvoltage on the *y*-axis. The gradual transitions towards different AoAs illustrate the continuity of the effect.

To more clearly illustrate the patterns we see in the ‘full effect’ plots, Fig 9 shows the effect over time on the ERP signal that our GAM model predicts for learners with an AoA of 10, 20 and 30, for both verb and gender violations (i.e. this is merely an alternative visualization of Panels A4 and B4 in Fig 8: AoA values are now represented by three separate plots for three AoA values, with microvoltage on the *y*-axis). These plots clearly illustrate the continuity of the effects, in the sense that the transitions towards different AoAs are gradual.

Finally, as indicated before, we tested two potential nuisance factors between subjects and items: test location and word frequency. Results of this model can be found in the Supporting Information: S2 File. We found no effect of test location Berlin on the general ERP patterns over time (*F* < 1), while Hamburg and Mainz did show small but significant effects (*F* = 8.77, *p* = .003; *F* = 6.13, *p* = .013). Inspection of the (visualizations of the) model output revealed, however, that the patterns described in the previous paragraph where highly similar (i.e. the time and AoA effects were unaffected). Differences between test locations are therefore likely to have resulted from the fact that AoA distributions were not balanced across locations. Word frequency did not significantly affect the AoA by time pattern in gender violations (*F* < 1) and there was only a minor effect on verb violations (*F* = 1.99, *p* = .048). Since neither of these nuisance factors had a large impact on the patterns reported above, they were not included in the final model reported in Table 4.

## Discussion

The goal of the current study was to investigate age of acquisition (AoA) effects on L2 grammar processing as revealed by the ERP signal. The objective was to perform an analysis that meets Birdsong’s [4] criteria for an appropriately fine-grained investigation of AoA effects by treating age of arrival as a continuous variable. Moreover, in order to capture the complexity of event-related potential (ERP) data, we did not limit the analysis to a particular time window, but instead looked at the (non-linear) ERP pattern over the entire time range in which effects might present themselves. This approach is arguably much more objective than pre-selecting a specific time span as is done in traditional approaches.

We also investigated whether the AoA effect was dependent on the grammatical structure under investigation. Since non-finite verbs are considered to be easy to acquire, we predicted to find little effect of AoA for these structures. Grammatical gender however, which is notoriously difficult for L2 learners, we expected to be particularly prone to AoA effects.

For violations in non-finite verb constructions a P600 was present across all AoAs, replicating earlier studies [30,31,33]. This finding demonstrates that native-like processing is possible for late learners, likely due to the regularity of the structure combined with similarity to participants’ L1. Previous studies using the same kind of non-finite verb constructions, found a delayed onset and/or delayed/reduced maximum of the P600 effect for late L2 learners (compared to natives) [30,33]. Another study however found learners’ P600 responses to non-finite verb violations to be very similar to natives’ [31]. In the current study, if anything, we find that the P600 has a slightly earlier onset and later offset as we get to higher AoAs, and that it reaches a higher maximum for later learners. It is difficult to pinpoint the reason for the differences between the four studies. A possible factor that might have contributed is a difference in the level of proficiency of the learners across studies, since there are some indications that the effect of a later/smaller P600 is stronger for lower proficiency learners [33]. The late learners in the current study (and in [31]) could simply be more proficient and therefore do not show a delay/reduction of the P600. The level of detail of the analysis may be another confounding factor: since the three previous studies used traditional approaches based on averaging with binning of an AoA group and ERP time window, we do not have as much information about AoA and latency/amplitude effects *within* the groups as we do in the current study. Despite this, it is clear that a P600 in response to non-finite verb violations is present for all learners in this study.

In contrast, gender violations showed a decreasing relationship between AoA and the size of the P600 effect: Whereas early learners with an AoA of up to around 20 are showing (diminishing) P600 effects, learners with later AoAs instead show a posterior negativity. The latter has not been found before in studies on L2 gender processing. Many previous studies have shown that it is in fact possible for late learners to show a native-like P600 effect in response to gender violations [29,46,47,49,50,52]. Some studies have found qualitative differences in the P600 response to gender violations for learners versus natives, in the form of a delayed/reduced effect [29,30,46,47]. The gradual transition from a native-like effect (a P600) for learners with lower AoAs to a very different brain response (a slightly earlier posterior negativity) for learners with higher AoAs in response to gender violations, has not been reported before. Regardless of the functional interpretation that one ascribes to ERP components such as the P600, a different component indicates that (partially) different groups of neurons are involved, in turn suggesting a different processing strategy. This transition from one processing strategy to another is reminiscent of the studies discussed in the introduction that report a gradual shift from an N400 to a P600 in the initial stages of L2 learning, thought to reflect a shift from the reliance on statistical dependencies to the induction of a generalized rule [23,24]. Although the current study investigates learners that have been immersed in the L2 environment for extended periods of time, and who should therefore be in the end rather than the initial stages of learning, a similar explanation may apply here. Grammatical gender might simply be a structure too complex for our late learners (coming from a significantly different L1 gender-system) to ever achieve fully native-like processing. However, further research is needed to investigate alternative explanations. The shift in processing strategies does not necessarily have to be caused by AoA (or maturation) itself, but could be due to a number of factors confounded with AoA, for example the fact that later learners have different experiences and learning contexts [67,68].

The fine-grained GAM analysis did not reveal any evidence of a clear discontinuity in the AoA curve. In both the verb and the gender condition, the (non-significant) AoA effects appeared to be linear, with no suggestion of a non-linearity. Furthermore, in interaction with time, the P600 for gender violations showed a steady decrease with an increase in AoA. This is clearly in contradiction with the conclusion we would draw from an ANOVA on the same dataset, which we also presented in this paper. The ANOVA would suggest the presence of a critical period before the age of 17, given that the learners with an AoA between 7 and 16 show a P600 for gender violations, but those in the group with an AoA of 17 and later do not. The absence of a discontinuity in the GAM analysis argues against the presence of a critical period. The discrepancy between the results of the two approaches clearly emphasizes the importance of Birdsong’s request for more granularity in studies of age effects.

The comparison between the traditional ANOVA and correlational analysis and our approach also reveals that the latter is more powerful. The fact that the GAM revealed no main effect of AoA, but only AoA effects in interaction with time, illustrates the need to include the explicit focus on the time course of the ERP signal in the analysis framework. Furthermore, the GAM analysis detected a negativity for late learners in response to gender violations, which did not become apparent in the ANOVA. To detect this effect another analysis would have been required, focusing on a different (subjectively selected) AoA group and time window. One other benefit of the GAM analysis compared to the ANOVA is the fact that we were able to rule out effects of potential nuisance factors (i.e. test location and word frequency) in the former.

Of course, there is still some subjectivity present in our analysis, due to our selection of a specific scalp region of interest. While taking into account the position of each electrode is possible within the generalized additive modeling framework (i.e. by creating a tensor of the interaction between *x* and *y* coordinates of the electrodes together with time), the computational requirements for such a model are high and can at present not be met when using the same amount of data for each electrode (i.e. the model described in this paper took about two months to fit). However, advances in the software and availability of high performance computing resources are likely to reduce the computational time required and so allow models that take into account data from individual electrodes in the future.

Another limitation of the present study is that we did not investigate other factors that might influence the performance of an L2 learner, such as years of exposure, learning environment, amount of daily L2 use and motivation. However, even though this might become computationally feasible in the near future, mulitcollinearity issues will likely make it difficult to tease apart some of these effects [55].

The final goal of our study was to compare AoA effects on an online (unconscious) neural measure to a behavioral measure that reflects conscious grammatical processing. During the EEG measurement, we therefore recorded participants’ perception of agreement violations in the form of end-of-sentence grammaticality judgments.

Our unconscious neural measure (ERPs) revealed two different processing strategies for early and late learners in dealing with gender violations. Regardless of the polarity, a difference between the brain response to correct and incorrect gender marked items indicates that both the early and the later learners are sensitive to the gender violations. This is consistent with the fairly good performance for learners in the offline gender assignment task (93% accuracy on average), meaning that learners are aware of the correct gender of the nouns. However, the results for the online grammaticality judgment task show that there is great variability in conscious recognition of gender errors in speech, when learners are under time pressure. As expected, Slavic learners, whose L1 does not have determiners, missed the ungrammaticality marked on determiners more often than the controls. This was particularly true for late learners, evidenced by the finding that AoA had a negative impact on accuracy.

Thus, we conclude that the alternative routes that the late learners have adopted to process gender agreement are computationally less efficient (possibly due to L1 influence), and make learners less confident about their conscious decisions under time pressure (see [69] and [34] for a similar argument). This might indicate that fully automatizing something that is so different from the L1 and lexically determined as syntactic gender, requires early exposure.

In conclusion, we show that the ERP signal in response to grammatical violations depends on the AoA of an L2 learner, as well as on the regularity of the structure under investigation. In (lexically determined) syntactic constructions different from the L1, we found a gradual change in processing strategies that varies by AoA, with a native-like effect for early learners and a less efficient neural processing strategy for later starters. These findings are based on the use of a novel method to analyze ERP data (generalized additive modeling, or GAM), taking into account individual differences, reducing subjectivity, and paying proper attention to the time course of the signal. A comparison with this approach to a more traditional analysis shows that the gradual shift in processing strategies that we observe with GAMs would not have surfaced in the latter type of analysis.

## Supporting Information

S1 File Data. (ZIP)

S2 File Analysis. (HTML)

S1 Table Materials. (PDF)
