# Supplementary material for: Age Effects in L2 Grammar Processing as Revealed by ERPs and How (Not) to Study Them
Source: PLoS One. 2015 Dec 18;10(12):e0143328. doi: 10.1371/journal.pone.0143328 (PMC4686163; doi:10.1371/journal.pone.0143328)
Supplement: S2 File — (HTML) [file pone.0143328.s002.html]

Data documentation, analysis script and output for Age effects in L2 grammar processing as revealed by ERPs and how (not) to study them


# Data documentation, analysis script and output for *‘Age effects in L2 grammar processing as revealed by ERPs and how (not) to study them’*

#### *Nienke Meulman1, Martijn Wieling1, Simone A. Sprenger1, Laurie A. Stowe1 & Monika S. Schmid1,2; 1University of Groningen, the Netherlands, 2University of Essex, UK*

#### *Generation date: November 07, 2015 - 11:36:46*

- 1. Packages and functions
- 2. Data sets
  - Grammaticality judgments
  - ERP data
- 3. Participant characteristics and scores on proficiency measures
  - Group characteristics
  - Overall L1 effects? (i.e. RU learners different from PL learners?)
  - Relationships between characteristics within individuals (correlations)
- 4. Behavioral results: grammaticality judgments
  - Distribution of accurate grammaticality judgments across participants:
  - Cross-linguistic similarity effects? (i.e. are learners more accurate when nouns have the same gender in their two languages?)
  - Mixed-effects logistic regression analysis of the accuracy of the answers to the grammaticality judgments
- 5. ERP results - Traditional analysis
  - Group comparison: ANOVA
  - Further inspection of the effect of AoA: correlation?
- 6. ERP results - GAM analysis
  - Model 1: The effects of Time and AoA for verb and gender violations
  - Model 2: Is the effect of gender violation significantly different from effect of verb violation?
  - Model 3: Model 1 including nuisance factors

### 1. Packages and functions

```
R.Version()$version.string
```

```
[1] "R version 3.1.2 (2014-10-31)"
```

```
library(mgcv); packageVersion("mgcv") # for gam analysis
```

```
[1] '1.8.4'
```

```
library(itsadug); packageVersion("itsadug") # for gam plotting
```

```
[1] '1.0.1'
```

```
library(lme4); packageVersion("lme4") # for lmer analysis
```

```
[1] '1.1.7'
```

```
library(ez); packageVersion("ez") # for anova analysis
```

```
[1] '4.2.2'
```

```
library(schoRsch); library(car); library(Hmisc); library(reshape)  # for anova output; levenes test; correlations
source('plotgrandav.R') # custom function
```

### 2. Data sets

```
load('gjdat.rda')
load('dat.rda')
# Note that the dataset for the grammaticality judgments does not contain the same ammount of trials as the ERP, since in the ERP data set trials with EEG artifacts have been removed, but these trials can still be used for the offline responses. And in the grammaticality judgments data the trails where no button press was registered were removed, but these still can be used for ERP analysis.
```

#### Grammaticality judgments

Legenda *gjdat* (12412 observations of 7 variables):  
1. Group : learners or natives  
2. Subject : subject number  
3. Word : target word (i.e. the word at the onset of which the ERP was measured)  
4. Correctness : grammaticality of the sentence, cor=grammatical; incor=ungrammatical  
5. Structure : GEN (gender) or VERB (non-finite verb) construction  
6. EndAcc : whether the participant responed correctly (1) or incorrectly (0) to the grammaticality judgment question for this item  
7. EndRT : the response time  
N.B. Practice trials and fillers have been removed from this data set, as well as trials where no button press was registered.

#### ERP data

Legenda *dat* (2390960 observations of 21 variables):  
1. uV : amplitude of the ERP signal measured in microVolt  
2. Time : time in milliseconds relative to the onset of the target word (one datapoint every 10 milliseconds)  
3. SeqStart : true when first data point of a new trial (used for autorcorrelation correction)  
4. TrialNr : postition of the sentence within the experiment  
5. Subject : subject number  
6. Group : learners or natives  
7. L1 : the first language of the learners (PL=Polisch, RU=Russian, DE=German)  
8. TestLocation : one of the three test locations: Hamburg (HH), Berlin (BLN) or Mainz (MZ)  
9. IsMale : 0=female, 1=male  
10. AoArr : the age of onset of acquisition (i.e. the age of arrival in the L2 country)  
11. AoExp : age of first exposure to the L2 in a classroom setting outside of the L2 country  
12. LoR : length of residence in the L2 country  
13. Age : age at testing  
14. Ctest : percentage of correct responses on the C-test (spelling errors were not penalized)  
15. GAtask : percentage of correct responses (i.e. a minimum of 2/3 instances of each item assigned correctly) on the post-EEG experiment gender assignment task  
16. Word : target word (i.e. the word at the onset of which the ERP was measured)  
17. Structure : GEN (gender) or VERB (non-finite verb) construction  
18. Correctness : grammaticality of the sentence, cor=grammatical; incor=ungrammatical  
19. Freq.log : the log transformed frequency of the target word (from Deutsches Referenzkorpus)  
20. EndAcc : whether the participant responed correctly (1) or incorrectly (0) to the grammaticality judgment question for this item  
21. List : which list was presented to this subject  
N.B. Practice trials and fillers have been removed from this data set, as well as trials with artifacts in the EEG data.

### 3. Participant characteristics and scores on proficiency measures

#### Group characteristics

Summary statistics for learners and natives and age of acquisition (AoA) distribution of the learners:

```
ppdat <- dat[!duplicated(dat$Subject),] # one row per subject
# learners
ldat <- ppdat[ppdat$Group=='learners', c(5:15)]; ldat <- droplevels(ldat)
summary(ldat[c(3:11)])
```

```
  L1     TestLocation IsMale     AoArr           AoExp            LoR             Age            Ctest      
 PL:15   BLN: 9       0:61   Min.   : 7.00   Min.   : 7.00   Min.   : 4.00   Min.   :18.00   Min.   :51.00  
 RU:51   HH :54       1: 5   1st Qu.:12.00   1st Qu.:10.00   1st Qu.: 8.00   1st Qu.:24.00   1st Qu.:74.00  
         MZ : 3              Median :19.00   Median :13.00   Median :10.00   Median :28.00   Median :84.00  
                             Mean   :17.68   Mean   :15.00   Mean   :11.26   Mean   :28.94   Mean   :80.86  
                             3rd Qu.:22.00   3rd Qu.:18.75   3rd Qu.:13.75   3rd Qu.:32.00   3rd Qu.:91.00  
                             Max.   :36.00   Max.   :32.00   Max.   :25.00   Max.   :53.00   Max.   :95.00  
     GAtask      
 Min.   : 72.92  
 1st Qu.: 89.84  
 Median : 94.27  
 Mean   : 93.31  
 3rd Qu.: 98.96  
 Max.   :100.00
```

```
par(mfrow=c(1,2))
plot(density(ldat$AoArr), main = "AoA density")
hist(ldat$AoArr, breaks=20, main = "AoA histogram")
```

```
table(ldat$AoArr) # first line shows AoAs, second line the nr. of participants for each AoA
```

```
 7  8  9 10 11 12 13 14 15 16 17 18 19 20 21 22 23 24 25 26 27 28 32 36 
 3  4  2  3  4  3  4  1  1  1  2  2  7  3  8  4  4  3  1  1  1  1  2  1
```

```
# natives
ndat <- ppdat[ppdat$Group=='natives', c(7:9, 13:15)]; ndat <- droplevels(ndat)
summary(ndat)
```

```
  L1     TestLocation IsMale      Age            Ctest           GAtask      
 DE:29   BLN: 1       0:19   Min.   :22.00   Min.   :86.00   Min.   : 98.96  
         HH : 7       1:10   1st Qu.:28.00   1st Qu.:93.00   1st Qu.:100.00  
         MZ :21              Median :37.00   Median :93.00   Median :100.00  
                             Mean   :37.79   Mean   :93.21   Mean   : 99.93  
                             3rd Qu.:46.00   3rd Qu.:95.00   3rd Qu.:100.00  
                             Max.   :58.00   Max.   :98.00   Max.   :100.00
```

Mann-Whitney U test for Age, Ctest and Gatask differences between groups:

```
wilcox.test(ppdat$Age ~ ppdat$Group)
```

```
    Wilcoxon rank sum test with continuity correction

data:  ppdat$Age by ppdat$Group
W = 498, p-value = 0.0002069
alternative hypothesis: true location shift is not equal to 0
```

```
wilcox.test(ppdat$Ctest ~ ppdat$Group)
```

```
    Wilcoxon rank sum test with continuity correction

data:  ppdat$Ctest by ppdat$Group
W = 197.5, p-value = 5.976e-10
alternative hypothesis: true location shift is not equal to 0
```

```
wilcox.test(ppdat$GAtask ~ ppdat$Group)
```

```
    Wilcoxon rank sum test with continuity correction

data:  ppdat$GAtask by ppdat$Group
W = 241.5, p-value = 1.427e-09
alternative hypothesis: true location shift is not equal to 0
```

#### Overall L1 effects? (i.e. RU learners different from PL learners?)

```
# C-test:
summary(ctestm <- lm(Ctest ~ L1 , data=ldat))
```

```
Call:
lm(formula = Ctest ~ L1, data = ldat)

Residuals:
    Min      1Q  Median      3Q     Max 
-30.216  -5.667   2.784   9.784  13.784 

Coefficients:
            Estimate Std. Error t value Pr(>|t|)    
(Intercept)   79.667      3.010  26.467   <2e-16 ***
L1RU           1.549      3.424   0.452    0.653    
---
Signif. codes:  0 '***' 0.001 '**' 0.01 '*' 0.05 '.' 0.1 ' ' 1

Residual standard error: 11.66 on 64 degrees of freedom
Multiple R-squared:  0.003187,  Adjusted R-squared:  -0.01239 
F-statistic: 0.2046 on 1 and 64 DF,  p-value: 0.6525
```

```
# GAtask:
gadat <- read.csv('GAtask_forR.csv', header=T, sep=',')
gadat <- melt(gadat, id=c("Trial","Word","Gender","EEGitemNr"))
names(gadat) <- c("Trial", "Word", "Gender", "EEGitemNr", "Subject", "Accuracy")
gadat <- gadat[!gadat$Subject %in% c("GN774","GN782","GL101","GL320","GL303","GL305"),] # remove subjects with too many EEG artifacts 
gadat <- with(gadat, aggregate(Accuracy, list(Subject, Word), mean))
colnames(gadat) <- c("Subject","Word","Accuracy")
gadat$Accuracy <- ifelse(gadat$Accuracy>0.66, 1, 0)
ppL1 <- ldat[c(1,3)]
gadat <- merge(gadat,ppL1,by=c("Subject"))
gadat$Accuracy <- as.factor(gadat$Accuracy)
summary(gaL1m <- glmer(Accuracy ~ L1 + (1|Subject), data=gadat, family='binomial'))
```

```
Generalized linear mixed model fit by maximum likelihood (Laplace Approximation) ['glmerMod']
 Family: binomial  ( logit )
Formula: Accuracy ~ L1 + (1 | Subject)
   Data: gadat

     AIC      BIC   logLik deviance df.resid 
  2517.9   2538.1  -1255.9   2511.9     6333 

Scaled residuals: 
    Min      1Q  Median      3Q     Max 
-8.9709  0.0796  0.1495  0.2722  0.6091 

Random effects:
 Groups  Name        Variance Std.Dev.
 Subject (Intercept) 2.502    1.582   
Number of obs: 6336, groups:  Subject, 66

Fixed effects:
            Estimate Std. Error z value Pr(>|z|)    
(Intercept)   3.9419     0.4621   8.530   <2e-16 ***
L1RU         -0.3909     0.5173  -0.756     0.45    
---
Signif. codes:  0 '***' 0.001 '**' 0.01 '*' 0.05 '.' 0.1 ' ' 1

Correlation of Fixed Effects:
     (Intr)
L1RU -0.873
```

```
# GramJudg task:
gjdat$Subject <- as.factor(ifelse(as.integer(as.character(gjdat$Subject))<699, 
                                  sub("^","GL",gjdat$Subject), sub("^","GN",gjdat$Subject))) # add GL/GN coding
gjdatL1 <- merge(gjdat,ppL1,by=c("Subject"))
gjdatL1$Accuracy <- as.factor(gjdatL1$EndAcc)
summary(gjL1m <- glmer(EndAcc ~ L1 + (1|Subject), data=gjdatL1, family='binomial'))
```

```
Generalized linear mixed model fit by maximum likelihood (Laplace Approximation) ['glmerMod']
 Family: binomial  ( logit )
Formula: EndAcc ~ L1 + (1 | Subject)
   Data: gjdatL1

     AIC      BIC   logLik deviance df.resid 
  7453.7   7474.8  -3723.9   7447.7     8336 

Scaled residuals: 
    Min      1Q  Median      3Q     Max 
-7.3576  0.1541  0.3560  0.6273  0.9401 

Random effects:
 Groups  Name        Variance Std.Dev.
 Subject (Intercept) 1.38     1.175   
Number of obs: 8339, groups:  Subject, 66

Fixed effects:
            Estimate Std. Error z value Pr(>|z|)    
(Intercept)   1.4878     0.3107   4.788 1.68e-06 ***
L1RU          0.3051     0.3547   0.860     0.39    
---
Signif. codes:  0 '***' 0.001 '**' 0.01 '*' 0.05 '.' 0.1 ' ' 1

Correlation of Fixed Effects:
     (Intr)
L1RU -0.875
```

#### Relationships between characteristics within individuals (correlations)

Relation between AoA and the other subject characteristics: age at testing, length of residence, L2 proficiency (as measured by the C-test) and offline gender knowledge (as measured by the gender assignment task):

```
cdat <- ppdat[ppdat$Group=='learners',]
cdat <- cdat[,c("AoArr","Age","LoR","Ctest","GAtask")]; cdat <- droplevels(cdat)
# transform skewed distributions
cdat$AoArr <- log(cdat$AoArr+1)
cdat$Age <- log(cdat$Age+1)
cdat$LoR <- log(cdat$LoR+1)
cdat$Ctest <- asin(cdat$Ctest/100)
cdat$GAtask <- asin(cdat$GAtask/100)
# correlation matrix:
print(rcorr(as.matrix(cdat))$r, digits=2)
```

```
       AoArr   Age   LoR Ctest GAtask
AoArr   1.00  0.71 -0.45 -0.43  -0.56
Age     0.71  1.00  0.27 -0.22  -0.25
LoR    -0.45  0.27  1.00  0.31   0.45
Ctest  -0.43 -0.22  0.31  1.00   0.55
GAtask -0.56 -0.25  0.45  0.55   1.00
```

```
# p values:
print(rcorr(as.matrix(cdat))$P, digits=6)
```

```
             AoArr         Age         LoR       Ctest      GAtask
AoArr           NA 2.16571e-11 0.000163701 2.76401e-04 1.27739e-06
Age    2.16571e-11          NA 0.029299546 7.77571e-02 3.91847e-02
LoR    1.63701e-04 2.92995e-02          NA 1.00258e-02 1.34480e-04
Ctest  2.76401e-04 7.77571e-02 0.010025754          NA 1.73935e-06
GAtask 1.27739e-06 3.91847e-02 0.000134480 1.73935e-06          NA
```

Multiple comparisons correction for AoA correlations reported in paper:

```
p <- c(2.16571e-11, 0.000163701, 2.76401e-04, 1.27739e-06)
round(p.adjust(p, "BH"), digits=5) # FDR correction
```

```
[1] 0.00000 0.00022 0.00028 0.00000
```

```
round(p.adjust(p, "bonferroni"), digits=5) # bonferroni correction
```

```
[1] 0.00000 0.00065 0.00111 0.00001
```

### 4. Behavioral results: grammaticality judgments

#### Distribution of accurate grammaticality judgments across participants:

```
myfun <- function(x) sum(x)/length(x) * 100
gj <- with(gjdat, aggregate(EndAcc, by = list(Group, Subject, Structure, Correctness), myfun))
names(gj) <- c("Group", "Subject", "Structure", "Correctness", "acc")
gj <- droplevels(gj)
gj$Group <- relevel(as.factor(gj$Group), "natives") # relevel so natives are first
gj$Structure <- relevel(as.factor(gj$Structure), "VERB") # verb first
par(mfrow=c(1,1), mar = c(9, 5, 4, 2) + 0.1)
boxplot(acc ~ Correctness * Structure * Group, data=gj, col="grey", main="Percentage of accurate reponses", ylab="%", las=2)
```

#### Cross-linguistic similarity effects? (i.e. are learners more accurate when nouns have the same gender in their two languages?)

```
gendat <- read.csv('PLandRUgenderEegItems.csv', header=T, sep=',') # gender similarity between German target nouns and Russian/Polish translation

# Grammaticality judgment task:
gjdatCLS <- merge(gjdatL1, gendat, by=c("Word")) 
gjdatCLS <- droplevels(gjdatCLS)
gjdatCLS$gensim <- ifelse(gjdatCLS$L1=="RU", as.character(gjdatCLS$simRUGE), as.character(gjdatCLS$simPLGE))
gjdatCLS$EndAcc <- as.factor(gjdatCLS$EndAcc)
gjdatCLS$gensim <- as.factor(gjdatCLS$gensim)
summary(gjCLSm <- glmer(EndAcc ~ gensim + (1|Subject), data=gjdatCLS, family='binomial'))
```

```
Generalized linear mixed model fit by maximum likelihood (Laplace Approximation) ['glmerMod']
 Family: binomial  ( logit )
Formula: EndAcc ~ gensim + (1 | Subject)
   Data: gjdatCLS

     AIC      BIC   logLik deviance df.resid 
  5378.7   5398.5  -2686.4   5372.7     5356 

Scaled residuals: 
    Min      1Q  Median      3Q     Max 
-6.1542 -0.9738  0.3380  0.6616  1.0628 

Random effects:
 Groups  Name        Variance Std.Dev.
 Subject (Intercept) 1.713    1.309   
Number of obs: 5359, groups:  Subject, 66

Fixed effects:
            Estimate Std. Error z value Pr(>|z|)    
(Intercept)  1.28858    0.16976   7.591 3.18e-14 ***
gensimsame   0.11773    0.06977   1.687   0.0915 .  
---
Signif. codes:  0 '***' 0.001 '**' 0.01 '*' 0.05 '.' 0.1 ' ' 1

Correlation of Fixed Effects:
           (Intr)
gensimsame -0.155
```

```
# Gender assignment task:
gadat2 <- merge(gadat, gendat, by=c("Word")) 
gadat2 <- droplevels(gadat2)
gadat2$gensim <- ifelse(gadat2$L1=="RU", as.character(gadat2$simRUGE), as.character(gadat2$simPLGE))
table(gadat2$L1, gadat2$gensim) # about 38% same gender words in Russian, about 44% same gender words in Polish
```

```
      dif same
  PL  795  645
  RU 3060 1836
```

```
gadat2$Accuracy <- as.factor(gadat2$Accuracy)
gadat2$gensim <- as.factor(gadat2$gensim)
summary(gaCLSm <- glmer(Accuracy ~ gensim + (1|Subject), data=gadat2, family='binomial'))
```

```
Generalized linear mixed model fit by maximum likelihood (Laplace Approximation) ['glmerMod']
 Family: binomial  ( logit )
Formula: Accuracy ~ gensim + (1 | Subject)
   Data: gadat2

     AIC      BIC   logLik deviance df.resid 
  2489.3   2509.5  -1241.6   2483.3     6333 

Scaled residuals: 
     Min       1Q   Median       3Q      Max 
-10.8291   0.0858   0.1298   0.2769   0.6796 

Random effects:
 Groups  Name        Variance Std.Dev.
 Subject (Intercept) 2.562    1.6     
Number of obs: 6336, groups:  Subject, 66

Fixed effects:
            Estimate Std. Error z value Pr(>|z|)    
(Intercept)   3.4323     0.2302  14.912  < 2e-16 ***
gensimsame    0.6482     0.1214   5.341 9.27e-08 ***
---
Signif. codes:  0 '***' 0.001 '**' 0.01 '*' 0.05 '.' 0.1 ' ' 1

Correlation of Fixed Effects:
           (Intr)
gensimsame -0.139
```

```
plogis(3.3779) # probability of accurate answer when different gender:
```

```
[1] 0.9670067
```

```
plogis(3.3779 + 0.8968) # probability of accurate answer when same gender:
```

```
[1] 0.9862748
```

#### Mixed-effects logistic regression analysis of the accuracy of the answers to the grammaticality judgments

We fit a mixed-effects logistic regression model with accuracy of the answer as the dependent variable (correct: 1 or incorrect: 0) assessing the importance of group (natives vs. learners), structure (gender vs. verb) and grammaticality (grammatical vs. ungrammatical). We included the maximal random effect structure supported by the data.

```
gjdat$EndAcc <- as.factor(gjdat$EndAcc)
gjdat$Structure <- as.factor(gjdat$Structure)
gjdat$Structure <- relevel(gjdat$Structure, "VERB") # verb as reference level
gjdat$Group <- relevel(gjdat$Group, "natives") # natives as reference level
gjdat$IsLearner = (gjdat$Group == 'learners')*1
# add AoAs:
aoa <- ppdat[1:66,c(5,10)]
gjdat$Subject <- as.factor(ifelse(as.integer(gjdat$Subject)<699, sub("^","GL",gjdat$Subject), 
                                  sub("^","GN",gjdat$Subject))) # add GL/GN coding
gjdat <- merge(gjdat, aoa, by="Subject", all=T)
table(gjdat$AoArr)
```

```
 7  8  9 10 11 12 13 14 15 16 17 18 19 20 21 22 23 24 25 26 27 28 32 36 
 3  4  2  3  4  3  4  1  1  1  2  2  7  3  8  4  4  3  1  1  1  1  2  1
```

```
gjdat[is.na(gjdat$AoArr),]$AoArr = 0 # for natives AoA coded as zero
```

```
summary(mgj0 <- glmer(EndAcc ~ Group*Structure*Correctness + (1+Structure*Correctness|Subject) + (1|Word), 
                      control=glmerControl(optimizer="bobyqa"), data=gjdat, family='binomial'))
```

```
Generalized linear mixed model fit by maximum likelihood (Laplace Approximation) ['glmerMod']
 Family: binomial  ( logit )
Formula: EndAcc ~ Group * Structure * Correctness + (1 + Structure * Correctness |      Subject) + (1 | Word)
   Data: gjdat
Control: glmerControl(optimizer = "bobyqa")

     AIC      BIC   logLik deviance df.resid 
  5888.0   6029.1  -2925.0   5850.0    12393 

Scaled residuals: 
     Min       1Q   Median       3Q      Max 
-12.5636   0.0601   0.1461   0.2596   4.2373 

Random effects:
 Groups  Name                          Variance Std.Dev. Corr             
 Word    (Intercept)                   0.19316  0.4395                    
 Subject (Intercept)                   0.96431  0.9820                    
         StructureGEN                  0.02856  0.1690    0.26            
         Correctnessincor              2.02040  1.4214    0.00 -0.62      
         StructureGEN:Correctnessincor 3.10436  1.7619   -0.29 -0.08  0.04
Number of obs: 12412, groups:  Word, 144; Subject, 95

Fixed effects:
                                            Estimate Std. Error z value Pr(>|z|)    
(Intercept)                                   4.8921     0.4324  11.313  < 2e-16 ***
Grouplearners                                -1.9248     0.4354  -4.420 9.85e-06 ***
StructureGEN                                  0.6362     0.4988   1.275   0.2022    
Correctnessincor                              1.0587     0.7040   1.504   0.1326    
Grouplearners:StructureGEN                   -0.6888     0.4757  -1.448   0.1476    
Grouplearners:Correctnessincor               -0.5795     0.6994  -0.829   0.4073    
StructureGEN:Correctnessincor                -1.8992     0.8334  -2.279   0.0227 *  
Grouplearners:StructureGEN:Correctnessincor  -1.1632     0.8353  -1.393   0.1637    
---
Signif. codes:  0 '***' 0.001 '**' 0.01 '*' 0.05 '.' 0.1 ' ' 1

Correlation of Fixed Effects:
            (Intr) Grplrn StrGEN Crrctn Gr:SGEN Grpl:C SGEN:C
Grouplernrs -0.899                                           
StructurGEN -0.604  0.514                                    
Crrctnssncr -0.462  0.415  0.343                             
Grplrn:SGEN  0.546 -0.559 -0.927 -0.318                      
Grplrnrs:Cr  0.419 -0.434 -0.302 -0.906  0.324               
StrctrGEN:C  0.282 -0.241 -0.578 -0.671  0.549   0.588       
Grpl:SGEN:C -0.243  0.230  0.522  0.585 -0.562  -0.603 -0.910
```

In the next model we have a look at the effect of AoA:

```
summary(mgj1 <- glmer(EndAcc ~ Structure:Correctness + AoArr:IsLearner:Structure:Correctness -1 + (1|Subject) + (1|Word), 
                      data=gjdat,family='binomial',control=glmerControl(optimizer="bobyqa")))
```

```
Generalized linear mixed model fit by maximum likelihood (Laplace Approximation) ['glmerMod']
 Family: binomial  ( logit )
Formula: EndAcc ~ Structure:Correctness + AoArr:IsLearner:Structure:Correctness -      1 + (1 | Subject) + (1 | Word)
   Data: gjdat
Control: glmerControl(optimizer = "bobyqa")

     AIC      BIC   logLik deviance df.resid 
  6465.6   6510.1  -3226.8   6453.6    12406 

Scaled residuals: 
     Min       1Q   Median       3Q      Max 
-23.9119   0.0466   0.1351   0.3242   3.1991 

Random effects:
 Groups  Name        Variance Std.Dev.
 Word    (Intercept) 0.1336   0.3655  
 Subject (Intercept) 4.2984   2.0733  
Number of obs: 12412, groups:  Word, 144; Subject, 95

Fixed effects:
                               Estimate Std. Error z value Pr(>|z|)    
StructureVERB:Correctnesscor     4.1567     0.2486  16.722  < 2e-16 ***
StructureGEN:Correctnesscor      4.0530     0.2361  17.165  < 2e-16 ***
StructureVERB:Correctnessincor   4.0474     0.2472  16.370  < 2e-16 ***
StructureGEN:Correctnessincor    1.3945     0.2249   6.199 5.67e-10 ***
---
Signif. codes:  0 '***' 0.001 '**' 0.01 '*' 0.05 '.' 0.1 ' ' 1

Correlation of Fixed Effects:
                     StrctrVERB:Crrctnssc StrctrGEN:Crrctnssc StrctrVERB:Crrctnssn
StrctrGEN:Crrctnssc  0.837                                                        
StrctrVERB:Crrctnssn 0.848                0.841                                   
StrctrGEN:Crrctnssn  0.854                0.926               0.858
```

```
plogis(4.59329 + (7*-0.08853)) # verb cor for AoA 7: 0.98 probability of accurate answer
```

```
[1] 0.9815411
```

```
plogis(4.59329 + (36*-0.08853)) # verb cor for AoA 36: 0.80 probability of accurate answer
```

```
[1] 0.8031675
```

```
plogis(3.95655 + (7*-0.20338)) # gen incor for AoA 7: 0.92 probability of accurate answer
```

```
[1] 0.9264156
```

```
plogis(3.95655 + (36*-0.20338)) # gen incor for AoA 36: 0.03 probability of accurate answer
```

```
[1] 0.03340319
```

### 5. ERP results - Traditional analysis

We calculate group averages over items, dividing the learners into early (AoA 7-16) and late (AoA 17-36) learners:

```
dat$AoAGroup <- ifelse(dat$AoArr %in% c(7:16), c("early learners"),
                       ifelse(dat$AoArr %in% c(17:36), c("late learners"), c("natives")))
dat$AoAGroup <- as.factor(dat$AoAGroup)
dat$AoAGroup <- relevel(dat$AoAGroup, "natives") # relevel so natives are first
dat$Structure <- relevel(as.factor(dat$Structure), "VERB") # verb first
# grand average plots (with 95% confidence intervals for the mean):
plot.grandaverage(dat) # custom function
```

#### Group comparison: ANOVA

We perform an anova analysis on the average amplitudes in the 500-1200 milliseconds time window:

```
# averaging over time window and items:
adat <- dat[dat$Time %in% c(500:1200),] 
adat <- with(adat, aggregate(uV, by=list(AoAGroup, Subject, Structure, Correctness), mean)) 
names(adat) <- c("AoAGroup","Subject","Structure","Correctness","uV")
# anova:
ezANOVA(data=adat, dv=uV, wid=Subject, within=.(Correctness, Structure), between=AoAGroup, detailed=F, type=3)
```

```
$ANOVA
                          Effect DFn DFd          F            p p<.05         ges
2                       AoAGroup   2  92   2.053059 1.341814e-01       0.017556326
3                    Correctness   1  92 116.407885 5.080914e-18     * 0.197730998
5                      Structure   1  92 116.681466 4.780986e-18     * 0.223838213
4           AoAGroup:Correctness   2  92   4.229267 1.749224e-02     * 0.017593757
6             AoAGroup:Structure   2  92   2.574865 8.164441e-02       0.012568161
7          Correctness:Structure   1  92  10.330875 1.804669e-03     * 0.019535378
8 AoAGroup:Correctness:Structure   2  92   2.322562 1.037439e-01       0.008879258
```

Because of the significant Group\*Correctness interaction, we do follow-up analysis per group:

```
for (grp in levels(as.factor(adat$AoAGroup))) {
  print(grp)
  print(ezANOVA(data=adat[adat$AoAGroup==grp,], dv=uV, wid=Subject, within=.(Correctness, Structure), detailed=F, type=3)) }
```

```
[1] "natives"
$ANOVA
                 Effect DFn DFd         F            p p<.05        ges
2           Correctness   1  28 83.685721 6.617263e-10     * 0.41543363
3             Structure   1  28 33.597202 3.170315e-06     * 0.19356785
4 Correctness:Structure   1  28  7.092863 1.268541e-02     * 0.02459276

[1] "early learners"
$ANOVA
                 Effect DFn DFd          F            p p<.05         ges
2           Correctness   1  25 40.9421506 1.065320e-06     * 0.167044671
3             Structure   1  25 26.2372448 2.717372e-05     * 0.246717914
4 Correctness:Structure   1  25  0.1501149 7.017046e-01       0.001374908

[1] "late learners"
$ANOVA
                 Effect DFn DFd        F            p p<.05        ges
2           Correctness   1  39 20.93170 4.737500e-05     * 0.10351950
3             Structure   1  39 71.82649 2.225523e-10     * 0.26383671
4 Correctness:Structure   1  39 11.99304 1.311144e-03     * 0.05276863
```

```
# FDR correction for mulitple testing:
round(p.adjust(c(7.017046e-01, 1.311144e-03, 1.268541e-02), "BH"), digits=3)
```

```
[1] 0.702 0.004 0.019
```

Only in late learners and natives the Correctness\*Structure interaction is significant. In these groups we therefore do follow-up analysis per structure:

```
for (grp in c("late learners", "natives")) {
  for (struc in levels(as.factor(adat$Structure))) {
    print(paste(grp, struc))
    test=adat[adat$AoAGroup==grp & adat$Structure==struc,]
    t_out(t.test(test$uV ~ test$Correctness))
    }
  }
```

```
[1] "late learners VERB"
                      Test                            Results
1 Welch Two Sample t-test: t(67.90) = -4.34, p < .001, d = NA
[1] "late learners GEN"
                      Test                            Results
1 Welch Two Sample t-test: t(70.94) = -1.16, p = .251, d = NA
[1] "natives VERB"
                      Test                            Results
1 Welch Two Sample t-test: t(50.44) = -6.08, p < .001, d = NA
[1] "natives GEN"
                      Test                            Results
1 Welch Two Sample t-test: t(47.35) = -7.39, p < .001, d = NA
```

```
# FDR correction:
round(p.adjust(c(0.2507, 4.867e-05, 2.034e-09, 1.586e-07), "BH"), digits=3)
```

```
[1] 0.251 0.000 0.000 0.000
```

Note: although group sizes are not equal, a levene’s test shows that variances are equal, so applying anovas here is justified:

```
# number of subjects per group:
myfunc <- function(x) length(unique(x)); aggregate(Subject ~ AoAGroup, adat, myfunc)
```

```
        AoAGroup Subject
1        natives      29
2 early learners      26
3  late learners      40
```

```
# standard deviation in each group:
sd(adat$uV[adat$AoAGroup=="early learners"]); sd(adat$uV[adat$AoAGroup=="late learners"]); sd(adat$uV[adat$AoAGroup=="natives"])
```

```
[1] 3.633292
```

```
[1] 4.083796
```

```
[1] 3.404438
```

```
leveneTest(uV ~ AoAGroup, data=adat)
```

```
Levene's Test for Homogeneity of Variance (center = median)
       Df F value Pr(>F)
group   2  0.9676 0.3809
      377
```

#### Further inspection of the effect of AoA: correlation?

In order to investigate the strength of the relation between AoA and the P600 effect, we performed a correlational analysis per individual in both AoA groups taken together. We plot the average magnitude of the difference wave (incorrect minus correct) in the 500-1200 ms time window per individual, and see if there is a correlation with AoA:

```
# averaging over time window and items:
adat <- dat[dat$Time %in% c(500:1200),] 
adat <- with(adat, aggregate(uV, by=list(Subject, AoArr, Structure, Correctness), mean)) # including AoA means natives dissapear
names(adat) <- c("Subject","AoArr","Structure","Correctness","uV")
# calculate difference between cor and incor conditions (=magnitude of the effect):
cdat <- adat[adat$Correctness == "cor",]
idat <- adat[adat$Correctness == "incor",]
dif <- merge(cdat, idat, by=c("Subject","AoArr","Structure"))
dif$dif <- dif$uV.y - dif$uV.x
dif <- dif[c("Subject","AoArr","Structure","dif")]

# plot magnitude by AoA, for VERB and GENDER separately
par(mfrow=c(1,2))
for (struc in levels(as.factor(dif$Structure))) {
  p <- dif[dif$Structure == struc,]
  with(p, plot(dif,AoArr, xlab=expression(paste("effect magnitude of difference wave in (",mu,"V",")")), 
               ylab="AoA", main=struc, font.main = 1))
  mtext(bquote(R^2 == .(round(summary(lm(dif~AoArr, p))$r.squared, digits=2))), cex=0.8)
  with(p, abline(lm(AoArr~dif), col="blue"))
  abline(v=0,lty=3)
  }
```

```
verb <- dif[dif$Structure == "VERB",]
summary(lm(dif~AoArr, verb))
```

```
Call:
lm(formula = dif ~ AoArr, data = verb)

Residuals:
    Min      1Q  Median      3Q     Max 
-8.8649 -2.5268 -0.9428  2.0939 18.4420 

Coefficients:
            Estimate Std. Error t value Pr(>|t|)  
(Intercept)  3.08822    1.57289   1.963   0.0539 .
AoArr        0.01846    0.08326   0.222   0.8253  
---
Signif. codes:  0 '***' 0.001 '**' 0.01 '*' 0.05 '.' 0.1 ' ' 1

Residual standard error: 4.498 on 64 degrees of freedom
Multiple R-squared:  0.0007673, Adjusted R-squared:  -0.01485 
F-statistic: 0.04914 on 1 and 64 DF,  p-value: 0.8253
```

```
rcorr(verb$dif, verb$AoArr)
```

```
     x    y
x 1.00 0.03
y 0.03 1.00

n= 66 


P
  x      y     
x        0.8253
y 0.8253
```

```
gender <- dif[dif$Structure == "GEN",]
summary(lm(dif~AoArr, gender))
```

```
Call:
lm(formula = dif ~ AoArr, data = gender)

Residuals:
    Min      1Q  Median      3Q     Max 
-7.0484 -1.9568 -0.0615  1.1822 13.3022 

Coefficients:
            Estimate Std. Error t value Pr(>|t|)    
(Intercept)  4.24542    1.14563   3.706 0.000442 ***
AoArr       -0.16317    0.06064  -2.691 0.009086 ** 
---
Signif. codes:  0 '***' 0.001 '**' 0.01 '*' 0.05 '.' 0.1 ' ' 1

Residual standard error: 3.276 on 64 degrees of freedom
Multiple R-squared:  0.1016,    Adjusted R-squared:  0.08759 
F-statistic:  7.24 on 1 and 64 DF,  p-value: 0.009086
```

```
rcorr(gender$dif, verb$AoArr)
```

```
      x     y
x  1.00 -0.32
y -0.32  1.00

n= 66 


P
  x      y     
x        0.0091
y 0.0091
```

### 6. ERP results - GAM analysis

In this section we will use generalized additive modelling (GAM) to assess the (possibly non-linear) effect of AoA on the non-linear ERP signal over time. We include the time-locked EEG signal per trial (i.e. per sentence per participant) of the learner data, in the full time range of -500 to 1400 ms before/after target onset. Grammaticality is converted into a binary variable (ungrammatical: 1 vs. grammatical: 0), to explicitly test for the non-linear difference between the two levels of grammaticality (i.e. to model the difference wave). To assess if a non-linear interaction between time and AoA was necessary, we fit a model that investigates the independent contribution of AoA vs. time on this difference, by means of decomposing the main effects of each of these factors and their pure interaction. The model also includes the time trends of individual participants per structure and level of grammaticality, and the time trends of individual items per level of grammaticality, to account for participant and item variation. Furthermore, each model includes a parameter to correct for the presence of autocorrelation in the residuals. As the response variable in our models (i.e. the ERP signal) is heavy-tailed, causing the residuals of our model to be non-normal, we fit the generalized additive model using the scaled-t family. As a consequence, the residuals follow the standard normal distribution.

#### Model 1: The effects of Time and AoA for verb and gender violations

```
dat$Group = as.factor(dat$Group)
dat = dat[!dat$Group == "natives",] # remove natives from dataset
dat = droplevels(dat)

# make ordered factors:
dat$Structure = as.factor(dat$Structure)
dat$Structure = relevel(dat$Structure, "VERB")

dat$IsVerbIncorrectO = (dat$Correctness == 'incor' & dat$Structure == 'VERB')*1
dat$IsVerbIncorrectO = as.ordered(dat$IsVerbIncorrectO)
contrasts(dat$IsVerbIncorrectO) = 'contr.treatment'

dat$IsGenderIncorrectO = (dat$Correctness == 'incor' & dat$Structure == 'GEN')*1
dat$IsGenderIncorrectO = as.ordered(dat$IsGenderIncorrectO)
contrasts(dat$IsGenderIncorrectO) = 'contr.treatment'

# make subject and item variation factors:
dat$SubjectCorStruc = interaction(dat$Subject, dat$Correctness, dat$Structure)
dat$WordCor = interaction(dat$Word, dat$Correctness)

rho = 0.8994937 # (from Gaussian model)
# Note: rho value is set to autocorrelation at lag 1 for the same model without rho throughout

source('bam.art.fit-v7.r') # coded by Natalya Pya

# Determine theta:
system.time(g0 <- gam(uV ~ 1, data=dat, method='REML', family=scat(rho=rho, AR.start=dat$SeqStart))) # 30 secs
theta0 <- g0$family$getTheta(TRUE) # we have tested this on smaller datasets, seems to be good estimate of theta for full model

# fit model:
gam1 <- bam(uV ~ s(Time,by=Structure) + s(AoArr,by=Structure) + ti(Time,AoArr,by=Structure) + #1
                 s(Time,by=IsVerbIncorrectO) + s(AoArr,by=IsVerbIncorrectO) + ti(Time,AoArr,by=IsVerbIncorrectO) + #2
                 s(Time,by=IsGenderIncorrectO) + s(AoArr,by=IsGenderIncorrectO) + ti(Time,AoArr,by=IsGenderIncorrectO) + #3
                 Structure + IsVerbIncorrectO + IsGenderIncorrectO + #4
                 s(Time, SubjectCorStruc,bs='fs',m=1) + s(Time,WordCor,bs='fs',m=1), #5
            data=dat, gc.level=2, method='fREML', family=art(theta=theta0, rho=rho), AR.start=SeqStart) 
            # line 1 = effect per structure, line 2 = effect of verb violation, line 3 = effect of gender violation, 
            # line 4 = fixed effects, line 5 = random wiggly curves
smrygam1 <- summary(gam1)
save(gam1,file='gam1.rda')
save(smrygam1,file='smrygam1.rda')
```

```
if (!file.exists('gam1.rda')) { 
    download.file('http://www.let.rug.nl/wieling/PaperPackages/MeulmanEtAl2015/gam1.rda',
                  'gam1.rda') # 1.4 GB
}
if (!file.exists('smrygam1.rda')) { 
    download.file('http://www.let.rug.nl/wieling/PaperPackages/MeulmanEtAl2015/smrygam1.rda',
                  'smrygam1.rda') # 374 MB
}
load('gam1.rda')
load('smrygam1.rda')
smrygam1
```

```
Family: Scaled t(5.214,4.685), rho=0.8994937 
Link function: identity 

Formula:
uV ~ s(Time, by = Structure) + s(AoArr, by = Structure) + ti(Time, 
    AoArr, by = Structure) + s(Time, by = IsVerbIncorrectO) + 
    s(AoArr, by = IsVerbIncorrectO) + ti(Time, AoArr, by = IsVerbIncorrectO) + 
    s(Time, by = IsGenderIncorrectO) + s(AoArr, by = IsGenderIncorrectO) + 
    ti(Time, AoArr, by = IsGenderIncorrectO) + Structure + IsVerbIncorrectO + 
    IsGenderIncorrectO + s(Time, SubjectCorStruc, bs = "fs", 
    m = 1) + s(Time, WordCor, bs = "fs", m = 1)

Parametric coefficients:
                    Estimate Std. Error t value Pr(>|t|)    
(Intercept)           0.9090     0.2898   3.136 0.001711 ** 
StructureGEN         -1.3840     0.3866  -3.580 0.000344 ***
IsVerbIncorrectO1     1.4992     0.4099   3.657 0.000255 ***
IsGenderIncorrectO1   0.4863     0.3624   1.342 0.179643    
---
Signif. codes:  0 '***' 0.001 '**' 0.01 '*' 0.05 '.' 0.1 ' ' 1

Approximate significance of smooth terms:
                                       edf   Ref.df      F  p-value    
s(Time):StructureVERB                6.590    7.674 12.826  < 2e-16 ***
s(Time):StructureGEN                 2.237    2.713 16.528 1.54e-09 ***
s(AoArr):StructureVERB               1.012    1.013  0.372    0.545    
s(AoArr):StructureGEN                1.289    1.302  0.467    0.544    
ti(Time,AoArr):StructureVERB         2.049    2.270  1.944    0.135    
ti(Time,AoArr):StructureGEN          1.971    2.124  1.785    0.164    
s(Time):IsVerbIncorrectO1            7.765    8.509 37.636  < 2e-16 ***
s(AoArr):IsVerbIncorrectO1           1.011    1.012  0.291    0.592    
ti(Time,AoArr):IsVerbIncorrectO1     3.879    3.957 10.236 3.59e-08 ***
s(Time):IsGenderIncorrectO1          7.793    8.599 18.288  < 2e-16 ***
s(AoArr):IsGenderIncorrectO1         1.060    1.063  2.194    0.135    
ti(Time,AoArr):IsGenderIncorrectO1   5.688    6.861  5.645 2.15e-06 ***
s(Time,SubjectCorStruc)            607.825 2368.000  1.900  < 2e-16 ***
s(Time,WordCor)                    250.227 2588.000  0.838  < 2e-16 ***
---
Signif. codes:  0 '***' 0.001 '**' 0.01 '*' 0.05 '.' 0.1 ' ' 1

R-sq.(adj) =  0.0434   Deviance explained = 3.27%
fREML = 5.064e+05  Scale est. = 1         n = 1613290
```

```
par(mfrow=c(4,3))
# VERB
fvisgam(gam1, plot.type='contour', view=c('Time','AoArr'), contour.col='black', color='topo', 
        zlim=c(-7,7), cond=list(IsVerbIncorrectO=1, IsGenderIncorrectO=0), rm.ranef=T,
        main='VERB: Full Time*AoA effect on difference')
```

```
Summary:
    * Structure : factor; set to the value(s): GEN. 
    * IsVerbIncorrectO : numeric predictor; set to the value(s): 1. 
    * IsGenderIncorrectO : numeric predictor; set to the value(s): 0. 
    * Time : numeric predictor; with 30 values ranging from -495.000000 to 1395.000000. 
    * AoArr : numeric predictor; with 30 values ranging from 7.000000 to 36.000000. 
    * SubjectCorStruc : factor; set to the value(s): GL105.cor.GEN. (Might be canceled as random effect, check below.) 
    * WordCor : factor; set to the value(s): aendern.cor. (Might be canceled as random effect, check below.) 
    * NOTE : The following random effects columns are canceled: s(Time,SubjectCorStruc),s(Time,WordCor)
```

```
plot.new()
plot.new()
constDiff = gam1$coef["IsVerbIncorrectO1"]
plot(gam1, shade=T, rug=F, select=7, seWithMean=T, shift=constDiff, ylim=c(6-constDiff,-4-constDiff), 
     main='s(Time):IsVerbIncorrect', ylab='uV'); abline(h=0)
plot(gam1, shade=T, rug=F, select=8, seWithMean=T, ylim=c(6,-4), 
     main='s(AoArr):IsVerbIncorrect', ylab='uV'); abline(h=0)
plot(gam1, rug=F, select=9, seWithMean=T, main='ti(Time,AoArr):IsVerbIncorrect',
     labcex=0.8, cex.lab=2, cex.axis=2, cex.main=1)
# GENDER
fvisgam(gam1, plot.type='contour', view=c('Time','AoArr'), contour.col='black', color='topo', 
        zlim=c(-7,7), cond=list(IsVerbIncorrectO=0, IsGenderIncorrectO=1), rm.ranef=T,
        main='GENDER: Full Time*AoA effect on difference')
```

```
Summary:
    * Structure : factor; set to the value(s): GEN. 
    * IsVerbIncorrectO : numeric predictor; set to the value(s): 0. 
    * IsGenderIncorrectO : numeric predictor; set to the value(s): 1. 
    * Time : numeric predictor; with 30 values ranging from -495.000000 to 1395.000000. 
    * AoArr : numeric predictor; with 30 values ranging from 7.000000 to 36.000000. 
    * SubjectCorStruc : factor; set to the value(s): GL105.cor.GEN. (Might be canceled as random effect, check below.) 
    * WordCor : factor; set to the value(s): aendern.cor. (Might be canceled as random effect, check below.) 
    * NOTE : The following random effects columns are canceled: s(Time,SubjectCorStruc),s(Time,WordCor)
```

```
plot.new()
plot.new()
constDiff = gam1$coef["IsGenderIncorrectO1"]
plot(gam1, shade=T, rug=F, select=10, seWithMean=T, shift=constDiff, ylim=c(6-constDiff,-4-constDiff), 
     main='s(Time):IsGenderIncorrect', ylab='uV'); abline(h=0)
plot(gam1, shade=T, rug=F, select=11, seWithMean=T, ylim=c(6,-4), 
     main='s(AoArr):IsGenderIncorrect', ylab='uV'); abline(h=0)
plot(gam1, rug=F, select=12, seWithMean=T, main='ti(Time,AoArr):IsGenderIncorrect', 
     labcex=0.8, cex.lab=2, cex.axis=2, cex.main=1)
```

Interpreting 2-dimensional smooth plots:

```
par(mfrow=c(2,3))
# verb
plot_smooth(gam1, view=c('Time'), cond=list(IsVerbIncorrectO=1, IsGenderIncorrectO=0, AoArr=10), 
            rm.ranef=T, main='Verb, AoA=10', rug=F, ylim=c(6,-5))
```

```
Summary:
    * Structure : factor; set to the value(s): GEN. 
    * IsVerbIncorrectO : numeric predictor; set to the value(s): 1. 
    * IsGenderIncorrectO : numeric predictor; set to the value(s): 0. 
    * Time : numeric predictor; with 30 values ranging from -495.000000 to 1395.000000. 
    * AoArr : numeric predictor; set to the value(s): 10. 
    * SubjectCorStruc : factor; set to the value(s): GL105.cor.GEN. (Might be canceled as random effect, check below.) 
    * WordCor : factor; set to the value(s): aendern.cor. (Might be canceled as random effect, check below.) 
    * NOTE : The following random effects columns are canceled: s(Time,SubjectCorStruc),s(Time,WordCor)
```

```
plot_smooth(gam1, view=c('Time'), cond=list(IsVerbIncorrectO=1, IsGenderIncorrectO=0, AoArr=20), 
            rm.ranef=T, main='Verb, AoA=20', rug=F, ylim=c(6,-5))
```

```
Summary:
    * Structure : factor; set to the value(s): GEN. 
    * IsVerbIncorrectO : numeric predictor; set to the value(s): 1. 
    * IsGenderIncorrectO : numeric predictor; set to the value(s): 0. 
    * Time : numeric predictor; with 30 values ranging from -495.000000 to 1395.000000. 
    * AoArr : numeric predictor; set to the value(s): 20. 
    * SubjectCorStruc : factor; set to the value(s): GL105.cor.GEN. (Might be canceled as random effect, check below.) 
    * WordCor : factor; set to the value(s): aendern.cor. (Might be canceled as random effect, check below.) 
    * NOTE : The following random effects columns are canceled: s(Time,SubjectCorStruc),s(Time,WordCor)
```

```
plot_smooth(gam1, view=c('Time'), cond=list(IsVerbIncorrectO=1, IsGenderIncorrectO=0, AoArr=30), 
            rm.ranef=T, main='Verb, AoA=30', rug=F, ylim=c(6,-5))
```

```
Summary:
    * Structure : factor; set to the value(s): GEN. 
    * IsVerbIncorrectO : numeric predictor; set to the value(s): 1. 
    * IsGenderIncorrectO : numeric predictor; set to the value(s): 0. 
    * Time : numeric predictor; with 30 values ranging from -495.000000 to 1395.000000. 
    * AoArr : numeric predictor; set to the value(s): 30. 
    * SubjectCorStruc : factor; set to the value(s): GL105.cor.GEN. (Might be canceled as random effect, check below.) 
    * WordCor : factor; set to the value(s): aendern.cor. (Might be canceled as random effect, check below.) 
    * NOTE : The following random effects columns are canceled: s(Time,SubjectCorStruc),s(Time,WordCor)
```

```
# gender
plot_smooth(gam1, view=c('Time'), cond=list(IsVerbIncorrectO=0, IsGenderIncorrectO=1, AoArr=10), 
            rm.ranef=T, main='Gender, AoA=10', rug=F, ylim=c(6,-5))
```

```
Summary:
    * Structure : factor; set to the value(s): GEN. 
    * IsVerbIncorrectO : numeric predictor; set to the value(s): 0. 
    * IsGenderIncorrectO : numeric predictor; set to the value(s): 1. 
    * Time : numeric predictor; with 30 values ranging from -495.000000 to 1395.000000. 
    * AoArr : numeric predictor; set to the value(s): 10. 
    * SubjectCorStruc : factor; set to the value(s): GL105.cor.GEN. (Might be canceled as random effect, check below.) 
    * WordCor : factor; set to the value(s): aendern.cor. (Might be canceled as random effect, check below.) 
    * NOTE : The following random effects columns are canceled: s(Time,SubjectCorStruc),s(Time,WordCor)
```

```
plot_smooth(gam1, view=c('Time'), cond=list(IsVerbIncorrectO=0, IsGenderIncorrectO=1, AoArr=20), 
            rm.ranef=T, main='Gender, AoA=20', rug=F, ylim=c(6,-5))
```

```
Summary:
    * Structure : factor; set to the value(s): GEN. 
    * IsVerbIncorrectO : numeric predictor; set to the value(s): 0. 
    * IsGenderIncorrectO : numeric predictor; set to the value(s): 1. 
    * Time : numeric predictor; with 30 values ranging from -495.000000 to 1395.000000. 
    * AoArr : numeric predictor; set to the value(s): 20. 
    * SubjectCorStruc : factor; set to the value(s): GL105.cor.GEN. (Might be canceled as random effect, check below.) 
    * WordCor : factor; set to the value(s): aendern.cor. (Might be canceled as random effect, check below.) 
    * NOTE : The following random effects columns are canceled: s(Time,SubjectCorStruc),s(Time,WordCor)
```

```
plot_smooth(gam1, view=c('Time'), cond=list(IsVerbIncorrectO=0, IsGenderIncorrectO=1, AoArr=30), 
            rm.ranef=T, main='Gender, AoA=30', rug=F, ylim=c(6,-5))
```

```
Summary:
    * Structure : factor; set to the value(s): GEN. 
    * IsVerbIncorrectO : numeric predictor; set to the value(s): 0. 
    * IsGenderIncorrectO : numeric predictor; set to the value(s): 1. 
    * Time : numeric predictor; with 30 values ranging from -495.000000 to 1395.000000. 
    * AoArr : numeric predictor; set to the value(s): 30. 
    * SubjectCorStruc : factor; set to the value(s): GL105.cor.GEN. (Might be canceled as random effect, check below.) 
    * WordCor : factor; set to the value(s): aendern.cor. (Might be canceled as random effect, check below.) 
    * NOTE : The following random effects columns are canceled: s(Time,SubjectCorStruc),s(Time,WordCor)
```

Model critisism:

```
par(mfrow=c(1,3))
#plot model residuals:
qqp(resid(gam1))
```

#### Model 2: Is the effect of gender violation significantly different from effect of verb violation?

```
dat$Group = as.factor(dat$Group)
dat = dat[!dat$Group == "natives",] # remove natives from dataset
dat = droplevels(dat)

# make ordered factors:
dat$Structure = as.factor(dat$Structure)
dat$Structure <- relevel(dat$Structure, "VERB")

dat$IsIncorrectO = (dat$Correctness == 'incor')*1
dat$IsIncorrectO = as.ordered(dat$IsIncorrectO)
contrasts(dat$IsIncorrectO) = 'contr.treatment'

dat$IsGenderIncorrectO = (dat$Correctness == 'incor' & dat$Structure == 'GEN')*1
dat$IsGenderIncorrectO = as.ordered(dat$IsGenderIncorrectO)
contrasts(dat$IsGenderIncorrectO) = 'contr.treatment'

# make subject and item variation factors:
dat$SubjectCorStruc = interaction(dat$Subject, dat$Correctness, dat$Structure)
dat$WordCor = interaction(dat$Word, dat$Correctness)

# gam2
rho = 0.8994944 # 22/10/14
system.time(gam2 <- bam(uV ~ s(Time,by=Structure) + s(AoArr,by=Structure) + ti(Time,AoArr,by=Structure) + #1
                        s(Time,by=IsIncorrectO) + s(AoArr,by=IsIncorrectO) + ti(Time,AoArr,by=IsIncorrectO) + #2
                        s(Time,by=IsGenderIncorrectO) + s(AoArr,by=IsGenderIncorrectO) + ti(Time,AoArr,by=IsGenderIncorrectO) + #3
                        Structure + IsIncorrectO + IsGenderIncorrectO + #4
                        s(Time, SubjectCorStruc,bs='fs',m=1) + s(Time,WordCor,bs='fs',m=1), #5
                      data=dat, gc.level=2, method='fREML', family=art(theta=theta0, rho=rho), AR.start=SeqStart)) 
                    # line 1 = effect per structure, line 2 = effect of violation, line 3 = is the effect of gender violation different 
                    # from effect of verb violation?, line 4 = fixed effects, line 5 = random wiggly curves
save(gam2,file='results/tdistribution/gam2.rda')
smrygam2 <- summary(gam2)
save(gam2,file='gam2.rda')
save(smrygam2,file='smrygam2.rda')
```

```
if (!file.exists('gam2.rda')) { 
    download.file('http://www.let.rug.nl/wieling/PaperPackages/MeulmanEtAl2015/gam2.rda',
                  'gam2.rda') # 1.4 GB
}
if (!file.exists('smrygam2.rda')) { 
    download.file('http://www.let.rug.nl/wieling/PaperPackages/MeulmanEtAl2015/smrygam2.rda',
                  'smrygam2.rda') # 355 MB
}
load('gam2.rda')
load('smrygam2.rda')
smrygam2
```

```
Family: Scaled t(5.214,4.685), rho=0.8994944 
Link function: identity 

Formula:
uV ~ s(Time, by = Structure) + s(AoArr, by = Structure) + ti(Time, 
    AoArr, by = Structure) + s(Time, by = IsIncorrectO) + s(AoArr, 
    by = IsIncorrectO) + ti(Time, AoArr, by = IsIncorrectO) + 
    s(Time, by = IsGenderIncorrectO) + s(AoArr, by = IsGenderIncorrectO) + 
    ti(Time, AoArr, by = IsGenderIncorrectO) + Structure + IsIncorrectO + 
    IsGenderIncorrectO + s(Time, SubjectCorStruc, bs = "fs", 
    m = 1) + s(Time, WordCor, bs = "fs", m = 1)

Parametric coefficients:
                    Estimate Std. Error t value Pr(>|t|)    
(Intercept)           0.9173     0.2892   3.172 0.001512 ** 
StructureGEN         -1.3751     0.3852  -3.570 0.000357 ***
IsIncorrectO1         1.4808     0.4080   3.629 0.000284 ***
IsGenderIncorrectO1  -1.0058     0.5429  -1.852 0.063962 .  
---
Signif. codes:  0 '***' 0.001 '**' 0.01 '*' 0.05 '.' 0.1 ' ' 1

Approximate significance of smooth terms:
                                       edf   Ref.df      F  p-value    
s(Time):StructureVERB                7.488    8.429 26.068  < 2e-16 ***
s(Time):StructureGEN                 1.785    2.140 19.278 2.19e-09 ***
s(AoArr):StructureVERB               1.002    1.003  0.368 0.544544    
s(AoArr):StructureGEN                1.290    1.304  0.474 0.541169    
ti(Time,AoArr):StructureVERB         3.660    3.789  5.131 0.000584 ***
ti(Time,AoArr):StructureGEN          2.158    2.319  2.310 0.089911 .  
s(Time):IsIncorrectO1                8.253    8.807 51.034  < 2e-16 ***
s(AoArr):IsIncorrectO1               1.002    1.002  0.273 0.601527    
ti(Time,AoArr):IsIncorrectO1         3.797    3.949  9.057 3.36e-07 ***
s(Time):IsGenderIncorrectO1          1.001    1.001 33.346 7.64e-09 ***
s(AoArr):IsGenderIncorrectO1         1.016    1.017  2.015 0.154833    
ti(Time,AoArr):IsGenderIncorrectO1   1.284    1.452  7.953 0.001903 ** 
s(Time,SubjectCorStruc)            620.437 2368.000  1.922  < 2e-16 ***
s(Time,WordCor)                    250.065 2588.000  0.838  < 2e-16 ***
---
Signif. codes:  0 '***' 0.001 '**' 0.01 '*' 0.05 '.' 0.1 ' ' 1

R-sq.(adj) =  0.0434   Deviance explained = 3.28%
fREML = 5.0639e+05  Scale est. = 1         n = 1613290
```

```
par(mfrow=c(1,3))
constDiff = gam2$coef["IsGenderIncorrectO1"]
plot(gam2, shade=T, rug=F, select=10, seWithMean=T, shift=constDiff, ylim=c(4-constDiff,-4-constDiff), 
     main='s(Time):IsGenderIncorrect', ylab='uV'); abline(h=0)
plot(gam2, shade=T, rug=F, select=11, seWithMean=T, ylim=c(4,-4), 
     main='s(AoArr):IsGenderIncorrect', ylab='uV'); abline(h=0)
plot(gam2, rug=F, select=12, seWithMean=T, main='ti(Time,AoArr):IsGenderIncorrect', 
     labcex=0.8, cex.lab=2, cex.axis=2, cex.main=1)
```

```
par(mfrow=c(1,3))
#plot model residuals:
qqp(resid(gam2))
```

#### Model 3: Model 1 including nuisance factors

```
dat$Group = as.factor(dat$Group)
dat = dat[!dat$Group == "natives",] # remove natives from dataset
dat = droplevels(dat)

# make ordered factors:
dat$Structure = as.factor(dat$Structure)
dat$Structure = relevel(dat$Structure, "VERB")

dat$IsVerbIncorrectO = (dat$Correctness == 'incor' & dat$Structure == 'VERB')*1
dat$IsVerbIncorrectO = as.ordered(dat$IsVerbIncorrectO)
contrasts(dat$IsVerbIncorrectO) = 'contr.treatment'

dat$IsGenderIncorrectO = (dat$Correctness == 'incor' & dat$Structure == 'GEN')*1
dat$IsGenderIncorrectO = as.ordered(dat$IsGenderIncorrectO)
contrasts(dat$IsGenderIncorrectO) = 'contr.treatment'

# make subject and item variation factors:
dat$SubjectCorStruc = interaction(dat$Subject, dat$Correctness, dat$Structure)
dat$WordCor = interaction(dat$Word, dat$Correctness)

rho = 0.8994966 # 02/11/14
system.time(gam3 <- bam(uV ~ s(Time,by=Structure) + s(AoArr,by=Structure) + ti(Time,AoArr,by=Structure) + #1
                        s(Time,by=IsVerbIncorrectO) + s(AoArr,by=IsVerbIncorrectO) + ti(Time,AoArr,by=IsVerbIncorrectO) + #2
                        s(Time,by=IsGenderIncorrectO) + s(AoArr,by=IsGenderIncorrectO) + ti(Time,AoArr,by=IsGenderIncorrectO) + #3
                        te(Time,Freq.log,by=CorStruc) + te(Time,by=TestLocation) + #4
                        Structure + IsVerbIncorrectO + IsGenderIncorrectO + CorStruc + TestLocation + #5
                        s(Time, SubjectCorStruc,bs='fs',m=1) + s(Time,WordCor,bs='fs',m=1), #6
                      data=dat, gc.level=2, method='fREML', family=art(theta=theta0, rho=rho), AR.start=SeqStart)) 
                    # line 1 = effect per structure, line 2 = effect of verb violation, line 3 = effect of gender violation, 
                    # line 4 = nuisance factors, line 5 = fixed effects, line 6 = random wiggly curves
save(gam3,file='results/tdistribution/gam3.rda')
smrygam3 <- summary(gam3)
save(gam3,file='gam3.rda')
save(smrygam3,file='smrygam3.rda')
```

```
if (!file.exists('gam3.rda')) { 
    download.file('http://www.let.rug.nl/wieling/PaperPackages/MeulmanEtAl2015/gam3.rda',
                  'gam3.rda') # 1.5 GB
}
if (!file.exists('smrygam3.rda')) { 
    download.file('http://www.let.rug.nl/wieling/PaperPackages/MeulmanEtAl2015/smrygam3.rda',
                  'smrygam3.rda') # 401 MB
}
load('gam3.rda')
load('smrygam3.rda')
smrygam3
```

```
Family: Scaled t(5.214,4.685), rho=0.8994966 
Link function: identity 

Formula:
uV ~ s(Time, by = Structure) + s(AoArr, by = Structure) + ti(Time, 
    AoArr, by = Structure) + s(Time, by = IsVerbIncorrectO) + 
    s(AoArr, by = IsVerbIncorrectO) + ti(Time, AoArr, by = IsVerbIncorrectO) + 
    s(Time, by = IsGenderIncorrectO) + s(AoArr, by = IsGenderIncorrectO) + 
    ti(Time, AoArr, by = IsGenderIncorrectO) + te(Time, Freq.log, 
    by = Structure) + te(Time, Freq.log, by = IsVerbIncorrectO) + 
    te(Time, Freq.log, by = IsGenderIncorrectO) + te(Time, by = TestLocation) + 
    Structure + IsVerbIncorrectO + IsGenderIncorrectO + TestLocation + 
    s(Time, SubjectCorStruc, bs = "fs", m = 1) + s(Time, WordCor, 
    bs = "fs", m = 1)

Parametric coefficients:
                    Estimate Std. Error t value Pr(>|t|)    
(Intercept)           1.1019     0.4038   2.729 0.006354 ** 
StructureGEN         -1.4519     0.3887  -3.735 0.000188 ***
IsVerbIncorrectO1     1.4395     0.4130   3.485 0.000492 ***
IsGenderIncorrectO1   0.5174     0.3635   1.423 0.154626    
TestLocationHH       -0.1278     0.3236  -0.395 0.692879    
TestLocationMZ       -0.5861     0.6148  -0.953 0.340427    
---
Signif. codes:  0 '***' 0.001 '**' 0.01 '*' 0.05 '.' 0.1 ' ' 1

Approximate significance of smooth terms:
                                            edf    Ref.df      F  p-value    
s(Time):StructureVERB                 6.585e+00 7.671e+00  9.497 1.39e-12 ***
s(Time):StructureGEN                  1.004e+00 1.006e+00  0.382  0.53794    
s(AoArr):StructureVERB                1.012e+00 1.013e+00  0.511  0.47593    
s(AoArr):StructureGEN                 1.421e+00 1.438e+00  0.228  0.72047    
ti(Time,AoArr):StructureVERB          2.191e+00 2.431e+00  2.575  0.06476 .  
ti(Time,AoArr):StructureGEN           2.313e+00 2.488e+00  2.136  0.10427    
s(Time):IsVerbIncorrectO1             7.755e+00 8.497e+00 19.190  < 2e-16 ***
s(AoArr):IsVerbIncorrectO1            1.010e+00 1.011e+00  0.291  0.59180    
ti(Time,AoArr):IsVerbIncorrectO1      3.877e+00 3.946e+00 10.822 1.28e-08 ***
s(Time):IsGenderIncorrectO1           7.827e+00 8.632e+00 17.678  < 2e-16 ***
s(AoArr):IsGenderIncorrectO1          1.032e+00 1.034e+00  2.327  0.12539    
ti(Time,AoArr):IsGenderIncorrectO1    5.217e+00 6.252e+00  6.222 1.12e-06 ***
te(Time,Freq.log):StructureVERB       2.856e+00 3.374e+00 10.070 5.06e-07 ***
te(Time,Freq.log):StructureGEN        4.811e+00 6.159e+00  2.905  0.00737 ** 
te(Time,Freq.log):IsVerbIncorrectO1   5.650e+00 7.448e+00  1.997  0.04771 *  
te(Time,Freq.log):IsGenderIncorrectO1 2.013e+00 2.024e+00  0.836  0.43384    
te(Time):TestLocationBLN              2.826e-03 4.816e-03  0.003  0.99688    
te(Time):TestLocationHH               1.004e+00 1.006e+00  8.772  0.00302 ** 
te(Time):TestLocationMZ               1.009e+00 1.016e+00  6.134  0.01296 *  
s(Time,SubjectCorStruc)               5.960e+02 2.366e+03  1.873  < 2e-16 ***
s(Time,WordCor)                       2.463e+02 2.584e+03  0.825  < 2e-16 ***
---
Signif. codes:  0 '***' 0.001 '**' 0.01 '*' 0.05 '.' 0.1 ' ' 1

Rank: 5213/5214
R-sq.(adj) =  0.0436   Deviance explained = 3.31%
fREML = 5.0635e+05  Scale est. = 1         n = 1613290
```

```
par(mfrow=c(2,3))
constDiff = gam3$coef["IsVerbIncorrectO1"]
plot(gam3, shade=T, rug=F, select=7, seWithMean=T, shift=constDiff, ylim=c(6-constDiff,-4-constDiff), 
     main='s(Time):IsVerbIncorrect', ylab='uV'); abline(h=0)
plot(gam3, shade=T, rug=F, select=8, seWithMean=T, ylim=c(6,-4), 
     main='s(AoArr):IsVerbIncorrect', ylab='uV'); abline(h=0)
plot(gam3, rug=F, select=9, seWithMean=T, main='ti(Time,AoArr):IsVerbIncorrect',
     labcex=0.8, cex.lab=2, cex.axis=2, cex.main=1)

constDiff = gam3$coef["IsGenderIncorrectO1"]
plot(gam3, shade=T, rug=F, select=10, seWithMean=T, shift=constDiff, ylim=c(6-constDiff,-4-constDiff), 
     main='s(Time):IsGenderIncorrect', ylab='uV'); abline(h=0)
plot(gam3, shade=T, rug=F, select=11, seWithMean=T, ylim=c(6,-4), 
     main='s(AoArr):IsGenderIncorrect', ylab='uV'); abline(h=0)
plot(gam3, rug=F, select=12, seWithMean=T, main='ti(Time,AoArr):IsGenderIncorrect', 
     labcex=0.8, cex.lab=2, cex.axis=2, cex.main=1)
```

```
par(mfrow=c(1,3))
#plot model residuals:
qqp(resid(gam3))
```
