# Supplementary material for: Age Effects in L2 Grammar Processing as Revealed by ERPs and How (Not) to Study Them
Source: PLoS One. 2015 Dec 18;10(12):e0143328. doi: 10.1371/journal.pone.0143328 (PMC4686163; doi:10.1371/journal.pone.0143328)
Supplement: S1 Table — (PDF) [file pone.0143328.s003.pdf]

Grammatical gender sentences
1. Vielleicht ist der/*das rote Apfel in die Kiste mit den Zitronen geworfen worden.
2. Nach der Schlägerei ist das/*der Auge des Angestellten von der Krankenschwester versorgt worden.
3. Leider ist der/*das Bart des Opernsängers vor ein paar Wochen abrasiert worden.
4. Glücklicherweise ist der/*das frühe Beginn der Konferenz nach hinten verschoben worden.
5. Weil die Schmerzen stärker wurden, ist das/*der kranke Bein der Rentnerin amputiert worden.
6. Trotz Bedenkzeit ist das/*der beste Beispiel von den Studenten nicht genannt worden.
7. In diesem Jahrzehnt ist der/*das steile Berg noch von niemandem bestiegen worden.
8. Damals ist der/*das Bericht über den Zwischenfall von den betroffenen Mitarbeitern erstellt worden.
9. Gerade ist das/*der Bett von den Handwerkern bei Ikea zusammengebaut worden.
10. Sobald die Gäste saßen, ist das/*der kühle Bier frisch vom Fass ausgeschenkt worden.
11. Vor kurzer Zeit ist das/*der hässliche Bild für mehrere Millionen Euro verkauft worden.
12. Während des Winters ist der/*das kalte Boden mit der Spitzhacke bearbeitet worden.
13. Mithilfe mehrerer Nachbarn ist der/*das schwere Brand nach kurzer Zeit erstickt worden.
14. Nach dem Diktat ist der/*das Brief von der Sekretärin sofort verschickt worden.
15. Mittlerweile ist das/*der verbrannte Brot vom Bäckermeister aus dem Ofen geholt worden.
16. Vermutlich ist das/*der dicke Buch von dem alten Mönch kopiert worden.
17. Weil plötzlich das Telefon klingelte, ist das/*der Ei in der heißen Pfanne vergessen worden.
18. Da alle Vasen voll waren, ist der/*das verrostete Eimer mit den Blumen gefüllt worden.
19. Heute ist das/*der spannende Ende des Theaterstücks von den Amateuren gut aufgeführt worden.
20. Anfangs ist der/*das Erfolg des Sportlers nur von wenigen beachtet worden.
21. Obwohl in der Innenstadt viele Polizisten sind, ist das/*der Fahrrad des Postboten geklaut worden.
22. Regelmäßig ist das/*der Feld mit dem Genmais von den Behörden kontrolliert worden.
23. Weil die Luft im Raum so schlecht war, ist das/*der Fenster zum Schulhof geöffnet worden.
24. Da Sturm angekündigt war, ist das/*der Fest am späten Nachmittag abgesagt worden.
25. Wahrscheinlich ist das/*der hohe Fieber von einem noch unbekannten Virus ausgelöst worden.
26. Inzwischen ist das/*der verdorbene Fleisch aus allen Supermärkten entfernt worden.
27. Da ein Gewitter aufzog, ist der/*das verspätete Flug aus London nach Frankfurt umgeleitet worden.
28. Weil die Mieterin beinahe hingefallen war, ist der/*das Flur nicht mehr gebohnert worden.
29. Wieder ist der/*das klare Fluss von der Chemiefabrik verschmutzt worden.
30. Weil der Gast nachher abreist, ist das/*der Frühstück für ihn eingepackt worden.
31. Da sich der Mann mehrere Zehen gebrochen hatte, ist der/*das Fuß eingegipst worden.
32. Mehrmals ist der/*das Garten für die internationale Tulpenschau genutzt worden.
33. Wegen der Finanzkrise ist das/*der Geld der Steuerzahler an Banken verschwendet worden.
34. Wegen des eingeschalteten Fernsehers ist das/*der Geräusch von der Frau überhört worden.
35. Obwohl das Publikum müde war, ist der/*das himmlische Gesang des Chors sehr bejubelt worden.
36. Wegen der Ferien ist das/*der Geschenk an den Lehrer erst nach seinem Geburtstag überreicht worden.
37. Für den Werbespot ist das/*der geschminkte Gesicht des Models viele Male fotografiert worden.
38. Zufällig ist das/*der Gespräch der Spione vom Geheimdienst belauscht worden.
39. Weil es so kalt war, ist das/*der heiße Getränk von den Wartenden dankbar angenommen worden.
40. In einem Tempel mitten im Dschungel ist das/*der Gold der Inka von den Forschern gefunden worden.
41. Auf dem Fußballplatz ist das/*der frische Gras von einer Truppe Gärtner gesät worden.
42. Vor der Trauung ist das/*der Haar der Braut mit Klammern hochgesteckt worden.
43. Erstmals ist der/*das Hafen für das Segelfestival im September eingeplant worden.
44. Zufällig ist der/*das lange Hals der Giraffe bei dem Sturz nicht schlimmer verletzt worden.
45. Obwohl keiner damit gerechnet hat, ist das/*der teure Handy unbeschädigt im Fundbüro abgegeben worden.
46. Anscheinend ist das/*der verfallene Haus in unserer Straße renoviert worden.
47. Damit ihr Sohn gut aussieht, ist das/*der Hemd von der Mutter gebügelt worden.
48. Liebevoll ist das/*der Herz von den Kindergartenkindern ausgemalt worden.
49. Vor Sonnenuntergang ist das/*der trockene Holz von den Indianern für das Lagerfeuer gesammelt worden.
50. Bei der Gala ist der/*das auffällige Hut der Schauspielerin von vielen bewundert worden.
51. Heute ist das/*der runde Kissen von meiner Schwester mit roten Herzen bestickt worden.
52. Obwohl die Arbeiter aufgepasst haben, ist das/*der Klavier beim Umzug beschädigt worden.
53. Weil der Frühling angefangen hat, ist das/*der kurze Kleid in das Schaufenster gehängt worden.
54. Weil der Patient kaum noch laufen konnte, ist das/*der Knie beim Arzt geröntgt worden.
55. Endlich ist der/*das alte Koffer mit den Löchern auf den Müll geworfen worden.
56. Bevor die Gäste ankamen, ist der/*das Korb mit frischem Obst gefüllt worden.
57. Vor dem Wettkampf ist der/*das Körper des Bodybuilders mit viel Öl eingeschmiert worden.
58. Leider ist der/*das Laden nach wenigen Wochen schon wieder geschlossen worden.
59. Infolge des schweren Tsunamis ist das/*der Licht in ganz Tokio abgeschaltet worden.

60. Wieder ist das/*der Lied der Franzosen beim Eurovision Song Contest zum Gewinner gekürt worden.
61. Weil die Geschäfte schlecht gehen, ist der/*das Lohn der Arbeiter erst gestern überwiesen worden.
62. Als die Kinder klein waren, ist das/*der scharfe Messer in einer verschlossenen Schublade aufbewahrt worden.
63. Weil die Wand steinhart war, ist der/*das Nagel völlig schief hineingeschlagen worden.
64. Solange das Meer noch nicht zugefroren war, ist das/*der Netz von den Fischern ausgeworfen worden.
65. Vor Weihnachten ist das/*der Postamt hinter dem Hauptbahnhof überfallen worden.
66. Da niemand das Auto kaufen wollte, ist der/*das Preis vom Händler gesenkt worden.
67. Versehentlich ist der/*das Rand des Kuchens von dem Lehrling abgeschnitten worden.
68. Ohne technisches Gerät ist der/*das saure Regen von den Klimaschützern analysiert worden.
69. Vorsichtig ist der/*das silberne Ring vom Juwelier mit Diamanten verziert worden.
70. Nach langem Üben ist der/*das schwierige Satz von sämtlichen Schülern richtig ausgesprochen worden.
71. Weil die Reise morgen beginnt, ist das/*der weiße Schiff von der Mannschaft beladen worden.
72. Vor dem Unwetter ist der/*das Schirm gerade noch rechtzeitig von der Terrasse geräumt worden.
73. Sicherheitshalber ist der/*das Schlüssel zum Tresor unter der Hecke vergraben worden.
74. Bei dem Erdbeben ist der/*das Schrank von allen Familienmitgliedern festgehalten worden.
75. Für die abendliche Party ist der/*das See von vielen hundert Lichtern angestrahlt worden.
76. Bei den Olympischen Spielen ist der/*das glorreiche Sieg von der russischen Sprinterin errungen worden.
77. Wie Experten erwartet haben, ist der/*das antike Spiegel bei der Auktion schnell versteigert worden.
78. In der zweiten Halbzeit ist das/*der wichtige Spiel wegen Nebels unterbrochen worden.
79. Durch den Transport ist der/*das Stein an der Unterseite beschädigt worden.
80. Beinahe ist der/*das dünne Stift von dem ungeschickten Mädchen kaputtgemacht worden.
81. Vor dem Seniorenheim ist der/*das Stock des älteren Herrn von den Jugendlichen versteckt worden.
82. Weil das Material nicht ankam, ist der/*das Stuhl erst letzte Woche fertiggestellt worden.
83. Weil die Sonne schien, ist der/*das Tisch auf der Terrasse für das Mittagessen gedeckt worden.
84. Da das Essen fertig war, ist der/*das heiße Topf neben dem Herd abgestellt worden.
85. Beim Elfmeterschießen ist das/*der entscheidende Tor von dem neuen Spieler geschossen worden.
86. Obwohl der Magier in die Zukunft blicken konnte, ist der/*das schlimme Unfall nicht verhindert worden.
87. Trotz Protesten ist das/*der harte Urteil gegen den Dieb vollstreckt worden.
88. Weil Sonntag war, ist der/*das komplizierte Vertrag nicht abgeschlossen worden.
89. Nach dem Protestmarsch ist der/*das Wald von den Umweltschützern besetzt worden.
90. Vergebens ist das/*der Wasser zum Löschen in das Tal gebracht worden.
91. Leider ist das/*der prächtige Wetter von niemandem rechtzeitig vorhergesagt worden.
92. Während der Kontrolluntersuchung ist der/*das faule Zahn ohne Betäubung gezogen worden
93. Minutenlang ist das/*der Zeichen von den Schmugglern am Leuchtturm wiederholt worden.
94. Bevor die Polizei eintraf, ist der/*das geheime Zettel von dem Betrüger vernichtet worden.
95. Obwohl wenig Zeit war, ist das/*der schmutzige Zimmer von der Putzfrau gründlich gereinigt worden.
96. Gestern ist der/*das defekte Zug mit einem Kran von den Gleisen gehoben worden.

Non-finite verb sentences
1. Vielleicht konnte der Grafiker die Tabelle noch ändern/*geändert, bevor die Zeitschrift in den Druck ging.
2. Leider hat die Rose diesen Herbst noch nicht geblüht/*blühen, obwohl wir sie jeden Tag gegossen haben.
3. Nach seiner Feier hat das Geburtstagskind allen gedankt/*danken, obwohl es nicht jeden Gast persönlich kannte.
4. Heute hat die Besprechung zu lange gedauert/*dauern, sodass alle Teilnehmer erst spät Feierabend machen konnten.
5. Gerade hat der Tänzer seine Partnerin so schnell gedreht/*drehen, dass sie ihr Gleichgewicht verlor.
6. Wieder musste ich den Senf mit Gewalt aus der Tube drücken/*gedrückt, weil kaum noch etwas darin war.
7. Vor dem Bewerbungsgespräch hat Manuel noch einmal geduscht/*duschen, um einen guten Eindruck zu hinterlassen.
8. In diesem Kurs darfst du im November dreimal fehlen/*gefehlt, ohne dass du Extraaufgaben machen musst.
9. Zuverlässig hat der Hirte die Schafe zu einer Quelle geführt/*führen, an der sie ihren Durst stillen konnten.
10. Zunächst wollte Frank die überraschende Nachricht nicht glauben/*geglaubt, aber dann freute er sich doch ein wenig.
11. Im Hochsommer will das Paar auf Schloss Neuschwanstein heiraten/*geheiratet, denn dort ist es so romantisch.
12. Vor wenigen Jahren noch hat mein Großvater seine Wohnung mit Kohle geheizt/*heizen, obwohl das sehr umständlich war.
13. Beim Abendessen habe ich minutenlang an einem Stück Schnitzel gekaut/*kauen, bis ich es schließlich ausspuckte
14. Leider konnte Matthias nicht auf den Felsen klettern/*geklettert, da es angefangen hatte zu regnen.
15. Bestimmt hat Peter bloß so ein aufwändiges Menü gekocht/*kochen, um Martina zu beeindrucken.
16. Trotz des Rabatts hat der Fernseher 500 Euro gekostet/*kosten, was ich viel zu viel finde.
17. Nur mit hohen Schuhen konnte Laura ihren Ehemann küssen/*geküsst, denn er war einen halben Meter größer als sie.
18. Während der Zirkusvorstellung mussten die Kinder die ganze Zeit lachen/*gelacht, da die Clowns so witzig waren.
19. Ohne Verspätung konnte der Helikopter landen/*gelandet, um die Kanzlerin zu der Ausstellung zu bringen.
20. In den 60er-Jahren hat meine Tante in Amerika gelebt/*leben, obwohl ihr Vater in Deutschland arbeitete.
21. Gewiss soll man Bananen nicht neben Äpfel legen/*gelegt, weil sie dann matschig werden.
22. Schon in ihrer Jugend hat meine Oma das Meer geliebt/*lieben, obwohl sie in den Bergen aufgewachsen ist.
23. Vor der ersten Stunde soll Paul sich beim Direktor melden/*gemeldet, da er einen Mitschüler geschlagen hat.
24. Erst wollte Christina die Wohnung am Stadtrand mieten/*gemietet, bis sie dann doch etwas schöner Gelegenes fand.
25. Mehrmals im Jahr sollte man seine Akten ordnen/*geordnet, um den Überblick zu behalten.
26. Inzwischen darf der kleine Junge seine Reisetasche selbst packen/*gepackt, auch wenn er meistens die Hälfte vergisst.
27. Hoffentlich hast du das Auto nicht in der Seitenstraße geparkt/*parken, denn dort ist überall absolutes Halteverbot.
28. In der kleinsten Größe hat Mona der Rock nicht gepasst/*passen, weil sie etwas zugenommen hat
29. Ohne Handschuhe sollte man diese Blumen nicht pflücken/*gepflückt, denn die Dornen sind sehr spitz.
30. Versehentlich ist der Luftballon geplatzt/*platzen, da mein Vater ihn zu rasch aufgeblasen hat.
31. Mehrmals hat der Beamte die Formulare geprüft/*prüfen, bevor er sie den Teilnehmern gab.
32. An jedem Wochentag musste Carola die Treppe putzen/*geputzt, obwohl die meisten Menschen den Lift nehmen.
33. Während des Gottesdienstes sollen die Kinder nicht reden/*geredet, um andere Besucher nicht zu stören.
34. Allein konnte Frieda die ganze Arbeit nicht schaffen/*geschafft, die ihr Chef ihr aufgebürdet hatte.
35. Früher haben viele Lebensmittel besser geschmeckt/*schmecken, weil nur natürliche Zutaten gebraucht wurden.
36. Während seiner Krankheit hat die Tochter für ihren Vater gesorgt/*sorgen, obwohl sie beruflich viel zu tun hatte.
37. Leider musst du für einen Urlaub in den USA etwas mehr sparen/*gespart, da die Flüge kaum billiger geworden sind.
38. Sonntags dürfen die Kleinen im Hinterhof spielen/*gespielt, bis es dunkel wird.
39. Jetzt darfst du meinen Bruder auf keinen Fall stören/*gestört, wenn du keinen Streit mit ihm möchtest.
40. Gestern ist die Katze unseres Nachbarn vom Hausdach gestürzt/*stürzen, ohne sich dabei wehzutun.
41. Nach dem Stadtrundgang hat sich die Gruppe getrennt/*trennen, sodass jeder in sein Hotel gehen konnte.
42. Vor dem Test musst du die Vokabeln noch einmal gut üben/*geübt, nachdem du vorhin so viele nicht wusstest.
43. Bestimmt hat der Pilot den Absprung nur gewagt/*wagen, weil keine Passagiere im Flugzeug waren.
44. Beim dritten Versuch hat Robin endlich die richtige Nummer gewählt/*wählen, unter der er den Babysitter erreichen konnte.
45. Auf dem Jakobsweg kann man wandern/*gewandert, bis man in Santiago di Compostela angekommen ist.
46. Während der Bootstour hat das Mädchen geweint/*weinen, da seine Puppe über Bord gefallen ist.
47. Zu meinem Geburtstag habe ich mir ein Kaninchen gewünscht/*wünschen, damit ich endlich ein Haustier habe.
48. Nach dem Abendessen wollte Mirco mit Karte zahlen/*gezahlt, aber das Restaurant nimmt nur Bargeld.
